# Machine Learning and Intelligent Diagnostics in Dental and Orofacial Pain Management: A Systematic Review

**DOI:** 10.1155/2021/6659133

**Published:** 2021-04-26

**Authors:** Taseef Hasan Farook, Nafij Bin Jamayet, Johari Yap Abdullah, Mohammad Khursheed Alam

**Affiliations:** ^1^Maxillofacial Prosthetic Service, Prosthodontic Unit, School of Dental Sciences, Universiti Sains Malaysia, Health Campus, Kelantan 16150, Malaysia; ^2^Division of Clinical Dentistry (Prosthodontics), School of Dentistry, International Medical University, Kuala Lumpur 57000, Malaysia; ^3^Craniofacial Imaging and Additive Manufacturing Laboratory, School of Dental Sciences, Universiti Sains Malaysia, Health Campus, Kelantan 16150, Malaysia; ^4^College of Dentistry, Jouf University, Sakaka, Saudi Arabia

## Abstract

**Purpose:**

The study explored the clinical influence, effectiveness, limitations, and human comparison outcomes of machine learning in diagnosing (1) dental diseases, (2) periodontal diseases, (3) trauma and neuralgias, (4) cysts and tumors, (5) glandular disorders, and (6) bone and temporomandibular joint as possible causes of dental and orofacial pain.

**Method:**

Scopus, PubMed, and Web of Science (all databases) were searched by 2 reviewers until 29^th^ October 2020. Articles were screened and narratively synthesized according to PRISMA-DTA guidelines based on predefined eligibility criteria. Articles that made direct reference test comparisons to human clinicians were evaluated using the MI-CLAIM checklist. The risk of bias was assessed by JBI-DTA critical appraisal, and certainty of the evidence was evaluated using the GRADE approach. Information regarding the quantification method of dental pain and disease, the conditional characteristics of both training and test data cohort in the machine learning, diagnostic outcomes, and diagnostic test comparisons with clinicians, where applicable, were extracted.

**Results:**

34 eligible articles were found for data synthesis, of which 8 articles made direct reference comparisons to human clinicians. 7 papers scored over 13 (out of the evaluated 15 points) in the MI-CLAIM approach with all papers scoring 5+ (out of 7) in JBI-DTA appraisals. GRADE approach revealed serious risks of bias and inconsistencies with most studies containing more positive cases than their true prevalence in order to facilitate machine learning. Patient-perceived symptoms and clinical history were generally found to be less reliable than radiographs or histology for training accurate machine learning models. A low agreement level between clinicians training the models was suggested to have a negative impact on the prediction accuracy. Reference comparisons found nonspecialized clinicians with less than 3 years of experience to be disadvantaged against trained models.

**Conclusion:**

Machine learning in dental and orofacial healthcare has shown respectable results in diagnosing diseases with symptomatic pain and with improved future iterations and can be used as a diagnostic aid in the clinics. The current review did not internally analyze the machine learning models and their respective algorithms, nor consider the confounding variables and factors responsible for shaping the orofacial disorders responsible for eliciting pain.

## 1. Introduction

Pain is a subjective sensation and has varying tolerance thresholds [1]. Orofacial pain has multiple origins and varying intensities. The pain may arise from exposed dentin (hypersensitivity pain) [2] or from carious infection of the dental pulp (pulpitis) [3]. Untreated dental pulp encourages the infection to spread through the root canals into the periodontal tissue (apical periodontitis) [4, 5] and may cause swelling, infection, and bone loss (periapical abscess) [6]. Periodontal tissue can also be painfully infected without carious activity (gingivitis and periodontitis) [7]. Maxillofacial fractures [8], as well as iatrogenic trauma/infection during dental restorative/endodontic treatment [2], may elicit varying levels of pain. Bone diseases [9], temporomandibular joint disorders [10], space infections [11], salivary gland disorders [12, 13], and sinusitis [14] elicit pain. Furthermore, neuralgia and secondary sensory nerve compression due to growing cysts and tumors can elicit severe pain [15, 16]. These conditions are categorized as common diseases and disorders that elicit dental and orofacial pain in the dental clinic [17].

The clinician's ability to diagnose such events swiftly and accurately is pivotal in successful patient management. However, various studies have shown that incorrect diagnoses are fairly common among clinicians in such situations [5, 6, 18]. While pain itself might not be reliably quantified, machine learning/artificial intelligence (AI) has been recently deployed to detect and quantify various diseases which elicit pain within the orofacial region to aid in accurate diagnostics and management.

AI and computerized support, although not new to healthcare, have lately received a lot of attention within the sphere of dentistry. These reviews covered their potential dental applications [19], success in detecting precancerous lesions and metastases [20], effectiveness in improving the quality of maxillofacial radiology [21], success in orthodontic treatment [22], and orthopedic rehabilitation [23], as well as concurrent application with virtual reality to decrease anxiety in young patients [24]. However, the aforementioned reviews did not systematically explore the current diagnostic capabilities of AI in identifying common orofacial diseases and disorders and/or the subsequently elicited pain [17].

Therefore, the current review was conducted and narratively synthesized to explore the influence of machine learning in the following diagnostic roles: (1) pain associated with dental diseases, (2) pain associated with periodontal diseases, (3) pain associated with trauma and neuralgias, (4) pain associated with cysts and tumors, (5) pain associated with glandular disorders, and (6) pain arising from bone and temporomandibular joint. The clinical effectiveness of machine learning, potential variations and probable causes, and human versus machine comparisons were also explored. The effectiveness of AI's influence was quantified using accuracy (ability to correctly differentiate disease from control), sensitivity (correctly identifying diseased subjects), specificity (correctly identifying disease-free subjects), and precision (repeated correct diagnoses) as appropriate.

## 2. Materials and Methods

### 2.1. Research Design

The study adhered closely to the Preferred Reporting Items for Systematic Reviews and Meta-Analyses for Diagnostic Test Accuracy (PRISMA-DTA) guidelines but followed a narration-based, qualitative approach to represent the included literature [25].

### 2.2. Eligibility Criteria

The following inclusion and exclusion criteria were developed for the current systematic review.

#### 2.2.1. Inclusion Criteria

1. Original articles describing the use of intelligent computer-guided decision-making to diagnose orofacial diseases that produce symptomatic pain in humans
2. Studies that incorporated diagnostic management of pain and inflammation using deep learning and intelligent decision-making systems within all specialties of dentistry
3. Studies of intelligent technologies for emotion and facial expression recognition applied in facial pain diagnostics and healthcare

#### 2.2.2. Exclusion Criteria

1. Literature demonstrating the application of expert systems, deep learning, and intelligent tools for anatomical and physiological morphology and radiomics quality analyses
2. Studies on intelligent systems used to detect precancerous or metastatic cancerous lesions, monitor surgically intervened malignancies, or assess the quality of life changes following tumor metastasis and chemo/radiation therapy
3. Editorials, reviews, book chapters, opinion letters, magazine issues, product advertisements, conference proceedings, social media and blog posts, and articles written in a foreign language without accompanying English translation

### 2.3. Specific Study Characteristics for Diagnostic Comparisons

Eligible and included studies that made human versus machine diagnostic comparisons were further screened according to the following criteria:

- Index test and evaluating parameters*:* the sensitivity and/or specificity of clinically trained machine learning models
- Reference standards: diagnostic accuracy of clinicians in identifying target conditions
- Target conditions*:* isolation of dental diseases that lead to symptomatic pain in the following conditions: dentinal, pulpal, periodontal, and alveolar inflammatory diseases; traumatic and cranial neuralgic disorders; odontogenic and nonodontogenic orofacial growths; orofacial glandular inflammation, obstruction, and impaired function; and facial bone and joint disorders

### 2.4. Information Source

All data were extracted from Scopus, PubMed, and Web of Science (all databases) by one clinician specializing in digital rehabilitation and one computing and imaging specialist. The Web of Science databases included the WoS Core collection, Current Contents Connect, Derwent Innovations Index, KCI Korean Journal Database, Medline, Russian Science Citation Index, and SciELO Citation Index. The data was extracted from 2020 backward with no lower limits. The final search was made in early November 2020.

### 2.5. Electronic Search Strategy

The strategy was specifically formulated using Boolean Logic (AND) and wildcards (^∗^) to allow for the same search terms to be applicable for all databases without requiring any modifications thereby maximizing data output [26]. The following combinations were used in the search:

  [ Big AND data AND dent^∗^ AND pain ]; [ Deep AND learning AND smart AND dent^∗^ ]; [ Expert AND system^∗^ AND dent ]; [ Expert AND system^∗^ AND maxill^∗^ AND pain ]; [ Machine AND learning AND dent^∗^ AND pain ]; [ Neural AND network AND dent^∗^ AND pain ]; [ Neural AND network AND maxill^∗^ AND pain ]; [ Generative AND adversarial AND dent^∗^ ]; [Fuzzy AND network AND dent^∗^]; [ Artificial AND intelligen^∗^ AND dent^∗^ AND pain ]; [ Artificial AND intelligen^∗^ AND caries AND pain ]; [ Intelligen^∗^ AND ulcer AND pain ]; [ Smart AND dent^∗^ AND pain ]; [ Comput^∗^ AND Intelligen^∗^ AND pain AND diagnos^∗^ AND dent^∗^ ]; [ Smart AND diagnos^∗^ AND dent^∗^ AND pain ]; [ Smart AND diagnos^∗^ AND facial AND pain ]; [ Intelligen^∗^ AND pain AND face ]; [ Intelligen^∗^ AND pain AND dent^∗^ ]; [ Intelligen^∗^ AND device^∗^ AND dent^∗^ AND pain ]; [ Intelligen^∗^ AND Sensor^∗^ AND diagnos^∗^ AND dent^∗^ AND pain ]; [ Electr^∗^ AND Sensor^∗^ AND diagnos^∗^ AND maxill^∗^ AND pain ]; [ Intelligen^∗^ AND biosens^∗^ AND oral ]; [ Artificial AND Somatosensor^∗^ AND facial ]; [ Intelligen^∗^ AND Somatosensor^∗^ AND dent^∗^ ]; [ intelligen^∗^ AND inflam^∗^ AND facial ]; [Tensor AND pain AND dent^∗^ ]; [ Comput^∗^ AND language AND inflam^∗^ AND face ]; [ Intelligen^∗^ AND oral AND carcinoma ]; [ Augment^∗^ AND reality AND dent^∗^ AND pain ]; [ Virtual AND dent^∗^ AND diagnos^∗^ AND pain ]; [ Artificial AND Intelligen^∗^ AND implant^∗^ AND pain ]; [ Deep AND learning AND maxil^∗^ AND surg^∗^ ]; [ Intelligen^∗^ AND ortho^∗^ AND pain AND dent^∗^ ]; [ Deep AND learning AND radio^∗^ AND oral ]; [ Deep AND learning AND radiol^∗^ AND pulp^∗^ ]; [ Deep AND learning AND radiol^∗^ AND periodon^∗^ ].

### 2.6. Study Selection and Data Collection Process

Titles were screened for duplicates using Endnote v8.2, and the remaining manuscripts were then screened by abstract based on predefined eligibility criteria. The articles excluded during abstract screening were documented along with the theme of the study and the reasons for exclusion. The level of agreement between the two reviewers was measured using the kappa coefficient, and all disagreements were resolved by a face-to-face meeting. Finally, full papers were read, and ineligible articles were removed with the reason for removal being noted.

### 2.7. Data Extraction

The following data were extracted from the methodology and result sections of the selected papers: quantifications related to dental pain and the machine learning classification models used to develop the intelligent system; the number and conditional characteristics of the training dataset that was used to train the intelligent system; the number of test data used to evaluate the newly trained system with possible human comparisons along with their subsequent learning outcomes; and finally, the clinician's specific role in training or validating the machine learning model which was also documented.

### 2.8. Diagnostic Accuracy Measures

Specificity (Sp) and sensitivity (Sn) were measured along with accuracy (Ac) and precision (Pr) data which were collected. All obtained values were standardized to 0.00–1.00, and normalized data were given a 1-point standard deviation [27]. The number of learning data (*n*^*L*^) and test data (*n*^*T*^) was also collected. No eligible papers were excluded for not presenting one or more of the aforementioned summary measures.

### 2.9. Risk of Bias and Applicability

Studies that made a direct comparison to clinicians as reference standards were assessed for bias and applicability. The appropriateness of the machine learning model was evaluated using the Minimum Information about Clinical Artificial Intelligence Modeling (MI-CLAIM) checklist [28]. The risk of bias among studies and possible inconsistencies in the comparison were assessed using Joanna Brigg's Institute Critical Appraisal for Diagnostic Test Accuracy (JBI-DTA) checklist [29]. The findings from the MI-CLAIM and JBI-DTA were then used to evaluate the quality of the diagnostic evidence produced in the studies by using the Cochrane GradePro (GRADE approach) [30].

### 2.10. Additional Syntheses

A meta-analysis was deemed inappropriate due to the substantial functional differences and clinical heterogeneity present across the various disease classifications and machine learning models.

## 3. Results

### 3.1. Study Selection

**Figure 1:**
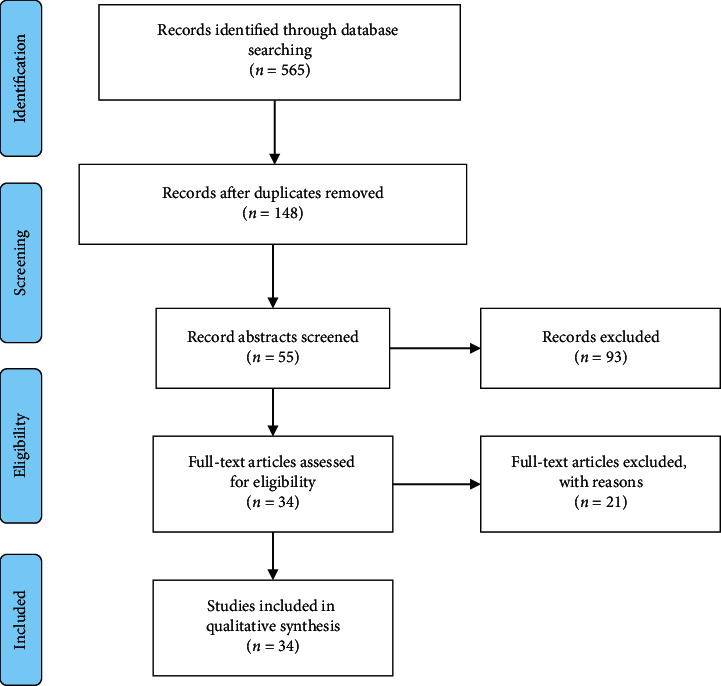
PRISMA flowchart of summary findings.

During the screening process, the reviewers had a fair agreement (*k* = 0.68) in the screening process. 34 articles were eventually selected for full paper reading based on eligibility criteria (Figure 1).

### 3.2. Study Characteristics and Individual Results

The study characteristics and their individual findings have been tabulated and presented as supplementary documents with this manuscript. The papers and tables are categorized into the following subsections: (1) pain associated with dental diseases [1–3, 31–37] (Supplementary Table S1), (2) pain associated with periodontal diseases [4–7, 18, 38–41] (Supplementary Table S2), (3) pain associated with trauma and neuralgias [8, 11, 16, 42] (Supplementary Table S3), (4) pain associated with cysts and tumors [15, 43, 44] (Supplementary Table S4), (5) pain associated with glandular disorders [12–14, 45] (Supplementary Table S5), and (6) pain arising from bone and temporomandibular joint [9, 10, 46–48] (Supplementary Table S6). The details of the articles excluded (and the entire study selection process) during systematic screening have been documented in Supplementary Material S7; Section 1.

### 3.3. Risk of Bias and Applicability

The current study of 34 published documents identified 8 articles [5, 6, 12–15, 31, 39] that made direct comparisons between the diagnostic accuracy of machine learning models and human clinicians. Of the 15 points evaluated from the MI-CLAIM checklist, all but one paper [39] scored over 13. JBI-DTA was assessed over 7 points where all papers scored 5 or more. Five of the 8 articles [5, 12–15, 39] could not avoid a case-control design as it was an integral part of the machine training process as found during MI-CLAIM. A “Range from studies” GRADE approach was undertaken to evaluate the collective diagnostic certainty of machine learning applicability. The GRADE approach suggested that a high certainty of diagnostic evidence for both positive and negative cases was present in machine learning. However, there were serious risks of collective bias and design inconsistencies among the cross-sectional cohorts that should be considered alongside the overall GRADE score. The conditions and explanations for all findings have been provided in Supplementary Material S7; Sections 2, 3, and 4.

### 3.4. Diagnostic Measure Comparisons

All 34 studies have been individually documented within Supplementary Tables S1to S6. Only the articles that made direct comparisons to clinicians have been documented in Table 1. All the studies mentioned in Table 1 have also been discussed in detail within the supplementary tables.

## 4. Discussion

### 4.1. Summary of Findings

The current review explored the clinical influence, effectiveness, limitations, and human comparison outcomes of machine learning. The findings of all 34 papers included within the systematic review have been discussed in the following subsections: (1) pain associated with dental diseases, (2) pain associated with periodontal diseases, (3) pain associated with trauma and neuralgias, (4) pain associated with cysts and tumors, (5) pain associated with glandular disorders, and (6) pain arising from bone and temporomandibular joint.

#### 4.1.1. Pain Associated with Dental Diseases

Real-time quantification of subjective dental pain demonstrated varying degrees of accuracy across multiple machine learning models when Hu et al. [1] attempted to detect (Ac = 0.80, Sn = 0.41, Sp = 0.89) and localize (Ac = 0.74, Sn = 0.54, Sp = 0.86) the source and intensity of dentin hypersensitivity pain arising from prefrontal and primary sensory cortices. The findings, in combination with Chattopadhyay's results [33], may contraindicate the implementation of an intelligent pain prediction system for perceived dental pain. Machine learning models based on clinically perceived pain produced less accurate outcomes for pulpal (Ac = 0.74–0.78, Sn = 0.48–0.71, Sp = 0.73–0.93) and periodontal diseases (Ac = 0.81, Sn = 0.78, Sp = 0.88), with the least accuracy (Ac = 0.64, Sn = 0.64, Sp = 0.96) for alveolar abscess [33]. Therefore, it can be argued that identifying the elusive source of dental pain is a more reliable estimate than quantifying pain as a symptom.

However, both proximal and periapical radiographs (Ac = 0.80, Sn = 0.75, Sp = 0.83) [31, 34] as well as histologically (Ac = 0.98, Pr = 0.98) trained models [2, 34] were able to reliably detect caries as a source for pain. While the aforementioned is considerably more efficient than clinicians (Ac = 0.71, Sn = 0.36, Sp = 0.91), dental specialists play an important role in training the machine from radiographs [3] or histological data [2]. Therefore, the prediction of the system may be directly dependent on the experience and agreement of the trainers.

Even periapical radiographs were capable of effectively (Ac = 0.82) detecting caries progression in posterior teeth [3]. Training dataset based on photographs (*n*^*L*^ = 425, Sn = 0.77–0.98, Sp = 0.84–0.96) [32] and photodetection (*n*^*L*^ = 24, Ac = 1.0) [37] produced varying outcomes when they were used to localize the progression of carious infiltration within the dentin layer [32]. This can be due to the funneling nature of caries progression as well as the small training datasets used. Many carious lesions, which visually appear negligible on the enamel surface, can funnel out within the dentin layer and cause sensitivity pain. Such factors were not considered in Rahman's study [37]. Researchers also attempted to provide camera-based intelligent solutions for end-users (patients). In such designs, video-learned systems (*n*^*L*^ = 10,080) produced reasonably reliable diagnoses of caries (Sn = 0.98, Sp = 0.93) and periodontitis (Sn = 0.97, Sp = 0.95) but were not very sensitive to painful microdefects like cracked teeth (Sn = 0.75, Sp = 0.99) [36].

#### 4.1.2. Pain Associated with Periodontal Diseases

The majority of the periodontal pain was associated with periodontal bone loss and root attachment loss which were, therefore, the primary quantification parameters [5]. Clinicians' experience was assumed to play a critical role in dictating the overall accuracy of radiographic differential diagnosis in machine learning. This assumption was confirmed by Chang et al. [38], Kim et al. [39], and Krois et al. [6] who found clinicians to make poorer diagnoses (Ac = 0.76, Sn = 0.78–0.92, Sp = 0.63–0.92) than their intelligent prodigies (Ac = 0.81, Sn = 0.77–0.81, Sp = 0.81–0.95). This was eventually reflected on the deep learning model as less accurate results with more variations were obtained contradicting Endres et al. [5], who found no significant correlation in their study. This could be due to the relatively low agreement (*k* = 0.48–0.52) between dental specialists [6, 18] in diagnosing a radiograph. Furthermore, Setzer's study [40] showed that the sensitivity of the machine in detecting periodontal diseases (Sn = 0.93, Sp = 0.88) was the same as the agreement between highly experienced specialists (*k* = 0.93). The clinicians themselves were inaccurate in diagnosing 31% of the time [5], and therefore, machine learning was deemed more specific. Periodontal conditions involved with larger bone defects [6] and indeciduous or crowded dentitions could affect predictive outcomes on panoramic radiographs (Sn = 0.84, Sp = 0.88, Pr = 0.81) [41]. Real-time/clinical machine learning, however, was less influenced by the operator's prowess [7] and heavily dependent on the accuracy of patient feedback (Ac = 0.82, Sn = 0.87, Sp = 0.76) during pain sensation [4].

#### 4.1.3. Pain Associated with Trauma and Neuralgias

Pain associated with root fractures is difficult to diagnose without a clear radiograph. With machine learning applied to clear panoramic radiographs, the intelligent system was less sensitive to localizing fractures on anterior teeth (Sn = 0.53, Pr = 0.88) as opposed to the posterior teeth (Sn = 0.70, Pr = 0.95) [8]. This was probably due to the vertebral shadow superimposing on the dental root anatomy [8, 18]. Trauma is often accompanied by painful swelling. Zhang et al. [11] demonstrated that a trained machine with a detailed patient history was able to accurately predict (Ac = 0.94–0.98) which patients were likely to experience painful swelling after tooth extractions.

McCartney et al. [16] and Limonadi et al. [42] designed and compared questionnaire-based intelligent systems to diagnose the source of facial pain. While the systems were accurate in diagnosing typical trigeminal neuralgia (Sn = 0.84–0.92, Sp = 0.83–0.84), it was observed that deep learning was not very sensitive to atypical neuralgias (Sn = 0.50–0.63, Sp = 0.94–0.95) [16, 42]. This is partly due to the idiopathic nature of certain diseases, which cause varying clinical symptoms including pain. Such variations can cause further disagreement in differential diagnoses among specialists, whose opinions are in turn used to train and validate the intelligent systems [6, 18]. The questionnaire-based method of deep learning hinges on the patients' ability to accurately report their conditions and pain intensity and was therefore may not be preferable for evaluating dental pain [1, 33].

#### 4.1.4. Pain Associated with Cysts and Tumors

Although most cysts, tumors, and other pathologic growths in the oral cavity are initially asymptomatic, growing lesions tend to elicit painful responses [15]. All the intelligent systems designed for tumor detection [15, 43, 44] were trained from panoramic radiographs by 2 expert radiologists. Watanabe et al. [44] carried out deep learning on larger (>10 mm) lesions, specifically radicular cyst lesions from panoramic radiographs (*n*^*L*^ = 330) where the authors found that the cortical thickness around the canine fossae and the maxillary sinus cavities drastically reduced prediction sensitivity (Sn = 0.46, Pr = 0.88 from Sn = 1.00, Pr = 0.92). Kwon's findings [43] agreed with Watanabe in that maxillary lesions were harder to predict. However, Kwon's results, which were based on a larger dataset (*n*^*L*^ = 946) and a pretrained neural network, saw comparatively better outcomes for radicular cysts (Ac = 0.96, Sn = 0.99, Sp = 0.83). This may indicate that the parameters used for machine learning in predicting oral tumors are more important than the experts who train the system. Deep learning produced better results for odontogenic keratocyst (Ac = 0.94, Sn = 0.70, Sp = 0.92, Pr = 0.63) when compared to diagnoses made by both surgeons (Sn = 0.67, Pr = 0.67) and general dentists (Sn = 0.64, Pr = 0.65) [15, 43]. This human-based discrepancy is probably due to the irregular shape and radiolucency of the tumor in respect to the rest of the mandibular anatomy. However, clinicians in Yang's study [15] were more sensitive (Sn = 0.36–0.45) to detecting well-defined ameloblastomas from radiographs than the trained machine (Sn = 0.33) [15].

#### 4.1.5. Pain Associated with Glandular Disorders

Maxillary sinusitis is an important differential diagnosis when evaluating the source of maxillary anterior pain. This can be done clinically by observing mucus discharge or through radiographs exhibiting glandular thickening within the sinus lining [14]. Kim et al. [45] and Murata et al. [14] showed machine learning to accurately detect sinusitis from both Water's view paranasal sinus (PNS) (Ac = 0.94, Sn = 0.89, SP = 0.99) and panoramic radiographs (Ac = 0.88, Sn = 0.86, Sp = 0.88). Deep learning outcomes from panoramic radiographs were comparable to diagnoses made by radiologists who had >20 years of experience (Ac = 0.90, Sn = 0.90, Sp = 0.89) and better agreement (*k* = 0.85) in diagnoses. [14, 45] Kim also demonstrated that when multiple trained virtual machines unanimously (*k* > 0.90) diagnose an image (majority decision analysis system), they produce accurate results (Ac = 0.94) [45] comparable to radiologists with over 30 years of diagnostic experience (Ac = 0.98) [12].

When assessing glandular disorders, radiologists demonstrated better agreement (*k* = 0.65) for disorders of visibly larger glands (parotid) as opposed to smaller glands (*k* = 0.51) obstructed by bony anatomy (submandibular gland) [13]. This deemed machine learning more sensitive to glandular anomalies but was also equally prone to making mistakes. Kise developed deep-learned systems to diagnose Sjogren's syndrome from both ultrasound imaging (*parotid gland*: Ac = 0.89, Sn = 0.90, SP = 0.89; *submandibular gland*: Ac = 0.84, Sn = 0.81, Sp = 0.87) [13] and computed tomography (Ac = 0.96, Sn = 1.00, SP = 0.92) [12]. The authors found that only clinicians with >30 years of experience were able to compete (Ac = 0.98, Sn = 0.99, Sp = 0.97) with the deep learning algorithm (Ac = 0.96, Sn = 1.00, Sp = 0.92) in diagnosing salivary gland disorders from 3D CT images [12]. The outcomes for clinicians were, however, substantially poorer when made to diagnose 2D radiographs (*parotid gland*: Ac = 0.77, Sn = 0.67, Sp = 0.86; *submandibular gland*: Ac = 0.72, Sn = 0.78, Sp = 0.66) [13]. Regardless, deep learning was shown to be a valuable diagnostic support for inexperienced clinicians (Ac = 0.77–0.84, Sn = 0.78, Sp = 0.75–0.89) [12, 14] to accurately diagnose gland-related orofacial pain.

#### 4.1.6. Pain Arising from Bone and Temporomandibular Joint

Temporomandibular joint (TMJ) disorders can cause severe pain for the patients [10]. Some of the painful disorders addressed by machine learning include joint osteoarthritis (Ac = 0.82, Sn = 0.83, Pr = 0.81) [47, 48], osteoporosis (Ac = 0.93, Sn = 0.97, Sp = 0.86) [10], reducible disk displacements (*unilateral*: Sn = 0.80, SP = 0.95; *bilateral*: Sn = 1.00, Sp = 0.89), and nonreducible disk displacements (*unilateral*: Sn = 0.69, SP = 0.91; *bilateral*: Sn = 0.37, Sp = 1.00) [46]. However, machine learning is still in its infancy primarily due to the complex diagnostic criteria required to confirm diseases like osteoarthritis [47]. The disease requires diagnostic confirmations from clinical, radiological, and serological findings and thereby complicate the machine learning procedure. Furthermore, Nam et al. [9] found pericoronitis and alveolar abscess to commonly (44%) mimic TMJ disorders which could be accurately differentiated (Ac = 0.96, SP = 0.99, Sn = 0.69) from true cases based on clinical symptoms using machine learning [9].

### 4.2. Limitations of the Study

At the time of conceptualization and data collection, the review protocol and study design were not registered with any databases that indexed ongoing reviews. Past literature suggests that such registrations can guard against reporting biases and validate the integrity of the published protocol [49]. In addition to the aforementioned, the current study was limited by several other factors. Firstly, foreign articles without a formal translation were not manually translated in order to prevent misinterpretation of the technical content and, therefore, may indicate a certain degree of publication bias. Secondly, this review did not internally analyze the different machine learning models and their respective algorithms and primarily focused on the clinical parameters. Furthermore, the current study did not account for the confounding variables and factors responsible for shaping the orofacial disorders responsible for eliciting pain. The difficulty in quantifying pain encouraged focusing on specific target conditions commonly, but not solely, responsible for pain. Finally, while the diagnostic comparisons yielded high certainty and low bias, the risk of bias and quality of evidence were not evaluated across the remaining 26 studies due to missing standard reference (human clinicians) comparison.

### 4.3. Conclusions and Future Recommendations

Machine learning in orofacial healthcare is still emerging and has shown modest results in diagnosing oral diseases. However, such technology is far from replacing clinicians in rendering healthcare and can possibly serve as an “add-on” to the existing diagnostic tools. Various workflows and methods exist for diagnosing dental diseases that can benefit from future crossovers and randomized trials on larger pools of patients in the future.

## Figures and Tables

**Table 1:** Summary outcomes of studies comparing diagnostic measures.

Author	Target condition definition	Testing sample size^a^	Index test outcomes^b^	Reference test outcomes^c^
Cantu et al. [31]	Extent and infiltration of proximal caries into dentinal tissue	141	Sn = 0.75, Sp = 0.83	Sn = 0.36, Sp = 0.91
Endres et al. [5]	Detect and classify periapical inflammation	102	Sn = 0.51	Sn = 0.51
Kise et al. [13]	Diagnose Sjogren syndrome in parotid and submandibular glands	40	*Parotid Gland* Sn = 0.90, Sp = 0.89 *Submandibular Gland* Sn = 0.81, Sp = 0.87	*Parotid Gland* Sn = 0.67, Sp = 0.86 *Submandibular Gland* Sn = 0.78, Sp = 0.66
Yang et al. [15]	Detect the presence of pathologic growth	181	Sn = 0.68	*Oral surgeons* Sn = 0.67 *General dentists* Sn = 0.64
Kim et al. [39]	Localize periodontal bone loss and classify apical lesions	800	Sn = 0.77, Sp = 0.95	Sn = 0.78, Sp = 0.92
Kise et al. [12]	Identify fatty degeneration within the salivary glands	100	Sn = 1.00, Sp = 0.92	*>3 years' experience* Sn = 0.99, Sp = 0.97 *<3 years' experience* Sn = 0.78, Sp = 0.89
Krois et al. [6]	To detect the extent of periodontal bone loss	353	Sn = 0.81, Sp = 0.81	Sn = 0.92, Sp = 0.63
Murata et al. [14]	Identify features of sinusitis	120	Sn = 0.86, Sp = 0.88	*>3 years' experience* Sn = 0.90, Sp = 0.89 *<3 years' experience* Sn = 0.78, Sp = 0.75

Sn: sensitivity; Sp: specificity; ^a^Testing samples: medical imaging data (radiographs/ultrasound/computed tomography); ^b^Index test: machine learning model; ^c^Reference test: human clinicians.

## Data Availability

All the supporting data have been provided as supplementary materials with the manuscript.

## Supplementary Materials

Supplementary Materials Supplementary Table S1: summary findings of literature for dental diseases. Supplementary Table S2: summary findings of literature for periodontal diseases. Supplementary Table S3: summary findings of literature for dental trauma and neuralgias. Supplementary Table S4: summary findings of the literature on cystic and neoplastic lesions. Supplementary Table S5: summary findings of the literature on glandular disorders. Supplementary Table S6: summary findings of the literature on bone and joint disorders. Supplementary Material S7.
