# Supplementary material for: Machine Learning and Intelligent Diagnostics in Dental and Orofacial Pain Management: A Systematic Review
Source: Pain Res Manag. 2021 Apr 26;2021:6659133. doi: 10.1155/2021/6659133 (PMC8093041; doi:10.1155/2021/6659133)
Supplement: Supplementary Materials — Supplementary Table S1: summary findings of literature for dental diseases. Supplementary Table S2: summary findings of literature for periodontal diseases. Supplementary Table S3: summary findings of literature for dental trauma and neuralgias. Supplementary Table S4: summary findings of the literature on cystic and neoplastic lesions. Supplementary Table S5: summary findings of the literature on glandular disorders. Supplementary Table S6: summary findings of the literature on bone and joint disorders. Supplementary Material S7. [file 6659133.f1.zip › 6659133.f1/Supplementary file S7 (updated on 7.2.21).pdf]

Supplementary material S7

Contents

Section 1: The search Process 2

Table S7.1: The search keywords used with generated search results in the databases 2

Table S7.2: Articles excluded during abstract screening and the reasons for exclusion 4

Table S7.3: Articles excluded after full paper read 13

Section 2: MI-CLAIM Checklist 15

Section 3: Cochrane GRADE 31

Section 4: JBI-DTA Checklist 33

Section 1: The search Process

Table S7.1: The search keywords used with generated search results in the databases

(Note: titles were screened from the generated search results and repeated titles were later removed in the duplicate removal process)

Search strings	Scopus	PubMed	Web of Sciences (All databases)	Interrogation date
Big AND data AND dent* AND pain	13	11	21	4th October 2020
Deep AND learning AND smart AND dent*	12	3	4	
Expert AND system* AND dent	31	279	25	
Expert AND system* AND maxill* AND pain	12	15	11	
Machine AND learning AND dent* AND pain	11	18	14	
Neural AND network AND dent* AND pain	25	98	74	12th October 2020
Neural AND network AND maxill* AND pain	2	10	6	
Generative AND adversarial AND dent*	10	12	11	
Fuzzy AND network AND dent*	55	17	51	
Artificial AND intelligen* AND dent* AND pain	11	15	16	
Artificial AND intelligen* AND caries AND pain	2	0	0	
Intelligen* AND ulcer AND pain	5	10	13	
Smart AND dent* AND pain	20	20	22	
Comput* AND Intelligen* AND pain AND diagnos* AND dent*	6	8	12	
Smart AND diagnos* AND dent* AND pain	4	2	7	
Smart AND diagnos* AND facial AND pain	3	6	8	
Intelligen* AND pain AND face	117	34	157	
Intelligen* AND pain AND dent*	41	47	48	
Intelligen* AND device* AND dent* AND pain	5	5	3	
Intelligen* AND Sensor* AND diagnos* AND dent* AND pain	2	3	4	
Electr* AND Sensor* AND diagnos* AND maxill* AND pain	19	57	32	
Intelligen* AND biosens* AND oral	6	9	12	
Artificial AND Somatosensor* AND facial	14	16	12	

Intelligen* AND Somatosensor* AND dent*	2	3	3	
intelligen* AND inflam* AND facial	12	34	7	
Tensor AND pain AND dent*	12	29	11	13th October 2020
Comput* AND language AND inflam* AND face	8	2	9	
Intelligen* AND oral AND carcinoma	39	50	71	
Augment* AND reality AND dent* AND pain	3	3	8	
Virtual AND dent* AND diagnos* AND pain	11	22	29	
Artificial AND Intelligen* AND implant* AND pain	14	6	18	
Deep AND learning AND maxil* AND surg*	28	57	28	
Intelligen* AND ortho* AND pain AND dent*	8	10	6	28th October 2020
Deep AND learning AND radio* AND oral	38	78	92	
Deep AND learning AND radiol* AND pulp*	2	4	3	29th October 2020
Deep AND learning AND radiol* AND periodon*	6	6	12	
**Total**	**609**	**999**	**860**	

Total Search results generated using Boolean and wildcards: 2468

Total titles extracted by both reviewers from the generated results: 565

Titles screened after duplicate removal: 148

Abstracts screened: 55

Full papers read: 34

Articles excluded during full paper reading: 21

Table S7.2: Articles excluded during abstract screening and the reasons for exclusion

Author	Title of the manuscript	Theme of the article	Reason for exclusion
Abbey, 1987	An expert system for oral diagnosis	Described a computerized database of dental patients and symptoms	text was unavailable
Maeda et al., 1987	An expert system for designing removable partial denture--the role of data base	-	Abstract was unavailable
Williams et al., 1987	A computer-controlled expert system for orthodontic advice	-	Abstract was unavailable
Jacobsen, 1991	Expert systems: computers in dentistry	-	Abstract was unavailable
Mackin et al., 1991	Artificial intelligence in the dental surgery: an orthodontic expert system, a dental tool of tomorrow	-	Abstract was unavailable
Masahiro, 1992	Clinical assessment of the knowledge base of an expert system for data analysis in laboratory medicine	Developed an expert system to detect haematological samples with iron-deficiency anemia	Did not concern orofacial pain management
Stheeman, 1992	Expert systems in dentistry. Past performance—future prospects	An educational review to instruct practitioners on the applications of expert system	Did not concern orofacial pain management
Davenport et al., 1997	Knowledge-based systems, removable partial denture design and the development of RaPiD	Applied expert systems to design partial dentures	Did not concern orofacial pain management
Van Waas et al., 1997	Construction of a clinical implant performance scale for implant systems with overdentures with the Delphi method	Obtained subjective scores of implant placement success for prosthetic rehabilitation	Focused on the development of a classification system
Ross et al., 1998	Computer applications in dental diagnosis	Provided a general overview of computer applications in dentistry	Did not concern orofacial pain management
Walther et al., 1998	Knowledge through documentation: from patients' data records to the basis of knowledge	Provided a general overview of computer applications in dentistry	Did not concern orofacial pain management
Gibbs et al., 1999	An algometer for intraoral pain tolerance measurements	Created an intraoral force analyzing device with a means of computerized data entry	Did not concern computer guided decision-making or pain management
Leader et al., 2001	Consequences of fiducial marker error on three-dimensional computer animation of the temporomandibular joint	Recorded the physiological jaw movement using sensor markers and computerized animations	Focused on anatomic and physiological analyses
Schleyer, 2001	Dental informatics: a cornerstone of dental practice	Provided a general overview of computer applications in dentistry	Did not concern orofacial pain management

Finkeissen et al. 2002	AIDA: Web agents to support dental treatment planning	Developed an artificial intelligence driven online tool to aid prosthetic rehabilitation	Did not concern orofacial pain management
Umar, 2002	Capabilities of computerized clinical decision support systems: the implications for the practicing dental professional	Provided a general overview of computer applications in Prosthetic dentistry	Did not concern orofacial pain management
Finkeissen et al. 2003	AIDA: web agents in dental treatment planning	enhanced the existing artificial intelligence driven online tool to further aid prosthetic rehabilitation	Did not concern orofacial pain management
Heo et al., 2003	Use of advanced imaging modalities for the differential diagnosis of pathoses mimicking temporomandibular disorders	Discussed preauricular pain and temporomandibular dysfunction diagnosis along with possible causes of misdiagnoses	Did not concern computer guided decision-making or pain management
Woda, 2003	Pain in the trigeminal system: from orofacial nociception to neural network modeling	-	Letter to the editor
Mendonca, 2004	Clinical decision support systems: perspectives in dentistry	Provided a general overview of computer applications in dentistry	Did not concern orofacial pain management
Gunter et al., 2005	Hydration level monitoring using embedded piezoresistive microcantilever sensors	Used piezo-driven sensors to analyse saliva for Sodium and humidity changes	Focused on anatomic and physiological analyses
Borra et al., 2007	Development of an open case-based decision-support system for diagnosis in oral pathology	Developed an expert system for healthcare education and record keeping	Did not concern orofacial pain management
Wierinck et al., 2007	Expert performance on a virtual reality simulation system	Dental experts were evaluated on their clinical expertise using virtual reality	Did not concern orofacial pain management
Prodan et al., 2008	Artificial intelligence for wound image understanding	Proposed an educational tool for e-learning	Conference proceeding
Olsen et al., 2009	Signal Processing and Machine Learning for Real-Time Classification of Ergonomic Posture with Unobtrusive On-body Sensors; Application in Dental Practice	Developed a wearable system to notify dental practitioners of harmful postures and thereby reduce ergonomics influenced pain	Conference proceeding
Solis et al., 2009	Development of Oral Rehabilitation Robot WAO-1R Designed to Provide Various Massage Techniques	Developed a machine to massage the maxillofacial region	Conference proceeding
Vikram, 2009	Decision support systems in dental decision making: an introduction	Provided a general overview of computer applications in dentistry	Did not concern orofacial pain management

Yagi et al., 2010	Decision-making system for orthodontic treatment planning based on direct implementation of expertise knowledge	Developed an expert system to predict when an extraction is indicated for orthodontic treatment	Did not concern orofacial pain management
Malcharek et al., 2011	Recordings of long-latency trigeminal somatosensory-evoked potentials in patients under general anaesthesia	Analysed the T-SSEP generated from trigeminal nerves	Focused on anatomic and physiological analyses
Clark et al. 2012	Autonomous virtual patients in dentistry: system accuracy and expert versus novice comparison	Compared the diagnostic accuracy between novice and expert practitioners	Did not concern computer guided decision-making or pain management
Exarchos et al., 2012	Multiparametric decision support system for the prediction of oral cancer reoccurrence	Studied an intelligent system that followed the progression and subsequent relapse of oral cancer	Did not concern orofacial pain management
Lee et al., 2012	Development of a decision making system for selection of dental implant abutments based on the fuzzy cognitive map	Created a system which classified tooth anatomy to predict the type of implant best suitable for rehabilitation	Focused on anatomic and physiological analyses
Milczewski et al., 2012	Force monitoring in a maxilla model and dentition using optical fiber Bragg gratings	Embedded optical sensors within orthodontic appliances to detect force application onto maxillary models	Did not concern computer guided decision-making or pain management
Salgueiro et al., 2013	An artificial neural network approach for predicting functional outcome in fibromyalgia syndrome after multidisciplinary pain program	An intelligent system was trained to detect cognitive neurological changes and predict reliable change at the time of discharge	Did not concern orofacial pain management
Sikka, 2014	Facial expression analysis for estimating pain in clinical settings	Used computer vision to detect facial changes and predict pain characteristics	Conference proceeding
Ye et al., 2014	A Bayesian Approach to Distinguishing Interdigitated Muscles in the Tongue from Limited Diffusion Weighted Imaging	Introduced trained systems into diffusion tensor imaging to minimize fiber tracking and resolve crossing fibers within tongue tissue	Did not concern orofacial pain management
Auconi et al., 2015	Prediction of Class III treatment outcomes through orthodontic data mining	Created a fuzzy clustering model to estimate individualized risks of failure in orthodontic treatment	Did not concern orofacial pain management
Russell et al., 2015	Toward Implementing Primary Care at Chairside: Developing a Clinical Decision Support System for Dental Hygienists	Created a clinical decision support system to address tobacco use, hypertension and diabetes screening along with dietary counselling	Did not concern orofacial pain management

Birdal et al., 2016	Automated lesion detection in panoramic dental radiographs	Used computerized tools to segment apical radiographs and use region growing to identify hard tissue changes	Did not concern orofacial pain management
Sannino et al., 2016	Dental and Biological Aspects for the Design of an Integrated Wireless Warning System for Implant Supported Prostheses: A Possible Approach	Proposed for the insertion of micro-displacement sensors within the implant prostheses to wirelessly report any warning signs	Describes preventive approaches to addressing prosthetic failure
Meng et al., 2016	Principal Component Analysis for Clustering Temporomandibular Joint Data	Developed a system to analyze the size of joint prostheses and accuracy of fit	Conference Proceeding
Anantharaman et al., 2017	Oro Vision: Deep Learning for Classifying Orofacial Diseases	Developed a mobile application to utilize camera and a pre-trained intelligent database, to capture and diagnose orofacial disease	Conference Proceeding
Miladinovic et al., 2017	Artificial intelligence in clinical medicine and dentistry	Provided a general overview of intelligent applications in dentistry	Did not concern orofacial pain management
Murata et al., 2017	Towards a Fully Automated Diagnostic System for Orthodontic Treatment in Dentistry	Developed an intelligent system to carry out morphological analyses prior to orthodontic treatment planning	Conference Proceeding
Thiam et al., 2017	Hierarchical combination of video features for personalised pain level recognition	Video data was fed to train the system to predict pain levels	Conference Proceeding
Wada et al., 2017	Altered structural connectivity of pain-related brain network in burning mouth syndrome-investigation by graph analysis of probabilistic tractography	Probabilistic tractography was used to study brain regions using diffusion tensor imaging. The study concluded that medial pain systems were altered from burning mouth syndrome	Did not concern computer guided decision-making or pain management
Yeh et al., 2017	A Wireless Monitoring System Using a Tunneling Sensor Array in a Smart Oral Appliance for Sleep Apnea Treatment	Created a smart device to monitor tongue pressure during sleeping and transmit the information via cloud	No implementations to orofacial healthcare
Yang, 2017	Neural network model based incremental oral ulcer predicting method, involves storing user recording daily data in incremental data table, and establishing modification ulcer pathological neural network model by incremental algorithm	-	Patent

Nugroho et al. 2018	On the Development of Smart Home Care: Application of Deep Learning for Pain Detection	Developed an intelligent interface that utilized smart home cameras to detect and predict facial pain	Conference proceeding
Omran et al., 2018	Decision support system for determination of forces applied in orthodontic based on fuzzy logic	Developed a fuzzy logic system which utilized Young's modulus and degree of pain to dictate the amount of force applied to the orthodontic wire	Conference Proceeding
Soar et al., 2018	Deep learning model for detection of pain intensity from facial expression	Developed a system to detect facial muscle movement and predict pain intensity	Conference Proceeding
Tolpadi et al., 2018	Inverse Biomechanical Modeling of the Tongue via Machine Learning and Synthetic Training Data	Utilized different activation patterns on virtual tongue data and measured amount of deformation	Focused on anatomic and physiological analyses
Wilkie et al., 2018	Identifying Potential Associations Between Patient Reported Outcomes and Normal Structure Dose Metrics in Head and Neck Cancer Patients Using a Big Data Approach	Analyzed patient documented symptoms with the therapy dose to deem which symptoms were dose-dependent	Did not concern orofacial pain management
Yang et al., 2018	IoT-Based Remote Pain Monitoring System: From Device to Cloud Platform	Proposed a wearable device to monitor pain intensity by analyzing facial surface electromyogram	No implementations to orofacial healthcare
Kheraif et al., 2019	Detection of dental diseases from radiographic 2d dental image using hybrid graph-cut technique and convolutional neural network	Produced a method to automatically separate teeth from background bone to classify dental anomalies	Focused on anatomic and physiological analyses
Ariji et al., 2019	Contrast-enhanced computed tomography image assessment of cervical lymph node metastasis in patients with oral cancer by using a deep learning system of artificial intelligence	Used deep learning to train the system to classify histologically positive and negative metastatic lymph nodes. The accuracy was evaluated by radiologists	Did not concern orofacial pain management
Askarian et al., 2019	Smartphone-Based Method for Detecting Periodontal Disease	Developed an intelligent system to use smartphone camera to detect and classify gingivitis	Conference Proceeding
Bhardwaj, 2019	Artificial Intelligence: Patient Care and Health Professional's Education	Provided a general overview of intelligent applications in dentistry	Did not concern orofacial pain management
Boyle et al., 2019	Using Virtual Human Technology to Examine Weight Bias and the	Used virtual models to evaluate healthcare	Did not concern computer guided decision-making or pain management

	Role of Patient Weight on Student Assessment of Pediatric Pain	students' perception of weight and chronic pain	
Bur et al., 2019	Machine learning to predict occult nodal metastasis in early oral squamous cell carcinoma	Trained the system to predict asymptomatic metastases accurately and thereby reduce clinical recommendations for neck dissection	Did not concern orofacial pain management
Fariza et al., 2019	Segmenting Tooth Components in Dental X-Ray Images Using Gaussian KernelBased Conditional Spatial Fuzzy C-Means Clustering Algorithm	Segmented teeth to accurately detect the different anatomical layers within	Focused on anatomic and physiological analyses
Gonella et al., 2019	A Cloud Fuzzy Logic Framework for Oral Disease Risk Assessment	Gathered knowledge from experts to create an expert system for clinicians in order to assess risks of dental caries and erosion	Conference Proceeding
Jurczyszyn, 2019	Differential diagnosis of leukoplakia versus lichen planus of the oral mucosa based on digital texture analysis in intraoral photography	Developed a system to differentiate between precancerous lesions using macro-photography and neural network guided differentiation	Did not concern orofacial pain management
Kesterke et al., 2019	Saving face: the role of artificial intelligence in evaluating craniofacial variation for the treatment of orofacial dysfunction	-	Abstract was unavailable
Moutselos et al., 2019	Recognizing Occlusal Caries in Dental Intraoral Images Using Deep Learning	Developed a caries prediction model based on ICDAS classification	Conference Proceeding
Naeini et al., 2019	An Edge-Assisted and Smart System for Real-Time Pain Monitoring	Utilized Biovid heat pain dataset to create an adaptive system to assess pain intensity at the Edge layer	Conference Proceeding
Puviarasi, 2019	Design and implementation of modernised dental chair using voice recognition control circuit	Developed an Arduino-based system to receive voice commands	Did not concern computer guided decision-making or pain management
Stark, 2019	Ensemble and Deep Learning for Real-time Sensors Evaluation of algorithms for real-time sensors with application for detecting brushing location	Utilized machine learning with sensor data to determine the tooth surface brushed	Conference Proceeding
Vinayahalingam et al., 2019	Automated detection of third molars and mandibular nerve by deep learning	Used machine learning to segment mandibular nerve from lower third molars on orthopantomograms	Focused on anatomic and physiological analyses
Xu et al., 2019	3D Tooth Segmentation and Labeling Using Deep Convolutional Neural Networks	Trained the system to detect tooth faces and	Focused on anatomic and physiological analyses

		segment the structures accordingly	
Yan et al., 2019	Tongue squamous cell carcinoma discrimination with Raman spectroscopy and convolutional neural networks	Developed an intelligent system to determine margin of tumor resection for operative procedures	Did not concern orofacial pain management
Brunenberg et al., 2020	External validation of deep learning-based contouring of head and neck organs at risk	Evaluated a commercially available deep learning contouring model	Focused on anatomic and physiological analyses
De Groof et al., 2020	Deep-learning system detects neoplasia in patients with barrett's esophagus with higher accuracy than endoscopists in a multistep training and validation study with benchmarking	Trained an intelligent system using endoscopic images of intestine to accurately identify Barrett's esophagus	Did not concern orofacial region
Di Scandalea et al., 2020	Deep Learning based diagnosis of Sjogren syndrome using In Vivo Confocal Microscopy	Segmented corneal nerve confocal microscopic images to diagnose ocular characteristics of Sjogren syndrome	Did not concern orofacial pain management
Fujima et al., 2020	Deep learning analysis using FDG-PET to predict treatment outcome in patients with oral cavity squamous cell carcinoma	Used deep learning on medical imaging to predict tumor stage and volumetric parameters	Did not concern orofacial pain management
Fukuda et al., 2020	Comparison of 3 deep learning neural networks for classifying the relationship between the mandibular third molar and the mandibular canal on panoramic radiographs	Compared 3 different models in the time taken and accuracy of segmenting mandibular third molars and mandibular canals	Did not concern orofacial pain management
Khan et al., 2020	Dental image analysis approach integrates dental image diagnosis	Provided a general overview of intelligent applications in dentistry	Did not concern orofacial pain management
Kim et al., 2020	Tooth segmentation of 3D scan data using generative adversarial networks	Generative adversarial network was used to recreate the occluded surfaces from intraoral scans	Did not concern orofacial pain management
Kuwada et al., 2020	Deep learning systems for detecting and classifying the presence of impacted supernumerary teeth in the maxillary incisor region on panoramic radiographs	Evaluated different intelligent systems in their ability to identify supernumerary teeth	Did not concern orofacial pain management
Laishram, 2020	Detection and classification of dental pathologies using faster-RCNN in orthopantomogram radiography image	Developed an algorithm to automatically classify teeth and some underlying pathologies	Conference Proceeding
Leite et al., 2020	Artificial intelligence-driven novel tool for tooth detection and	Ground truth was set up by radiologists which served as training models for the	Did not concern orofacial pain management

	segmentation on panoramic radiographs	intelligent system which aimed to classify teeth accurately	
Leite et al., 2020	Radiomics and Machine Learning in Oral Healthcare	Provided a general overview of intelligent applications in dental radiology	Did not concern orofacial pain management
Liang et al., 2020	OralCam: Enabling Self-Examination and Awareness of Oral Health Using a Smartphone Camera	Developed a deep learning model to analyze oral photographs and detect common oral diseases	Conference Proceeding
Mieronkoski et al., 2020	Developing a pain intensity prediction model using facial expression: A feasibility study with electromyography	Used electromyography to evaluate which facial muscles were most triggered during painful stimuli.	Focused on anatomic and physiological analyses
Nagi et al., 2020	Clinical applications and performance of intelligent systems in dental and maxillofacial radiology: A review	Provided a general overview of intelligent applications in dental radiology	Did not concern orofacial pain management
Rekow, 2020	Digital dentistry: The new state of the art—Is it disruptive or destructive?	Provided a general overview of computer applications in dentistry	Did not concern orofacial pain management
Robin et al., 2020	Recording of bruxism events in sleeping humans at home with a smart instrumented splint	Developed an ambulatory system to record clenching and masticatory force	Did not concern computer guided decision-making or pain management
Singh, 2020	Numbering and Classification of Panoramic Dental Images Using 6-Layer Convolutional Neural Network	Analyzed the accuracy of tooth anatomy classification from radiographs	Focused on anatomic and physiological analyses
Singh, 2020	Decision Support System for Black Classification of Dental Images Using GIST Descriptors	Developed a system to categorize dental caries according to Black's classification	Conference Proceeding
Salehi et al., 2020	Optimization methods for deep neural networks classifying OCT images to detect dental caries	Tested the efficiency of various optimization models in detecting dental caries	Conference Proceeding
Tonetti et al., 2020	Self-reported bleeding on brushing as a predictor of bleeding on probing: Early observations from the deployment of an internet of things network of intelligent power-driven toothbrushes in a supportive periodontal care population	Designed a smart toothbrush to record events of gingival bleeding during brushing	Did not concern computer guided decision-making or pain management
Yoshida, 2020	Accuracy of online symptom checkers for diagnosis of orofacial pain and oral medicine disease	Evaluated the prediction accuracy of online, consumer accessible, expert systems	Did not concern orofacial pain management

Yuan et al., 2020	Personalized design technique for the dental occlusal surface based on conditional generative adversarial networks	Used an intelligent model to design occlusal surface of virtual crowns	Did not concern orofacial pain management

Table S7.3: Articles excluded after full paper read

Author	Title of the manuscript	Reason for exclusion
White, 1996	Decision-support systems in dentistry	Full paper not available
Koch, 2003	Designing clinically useful systems: examples from medicine and dentistry	Did not concern computer guided decision-making or pain management or pain management
Ahlers, 2005	Evidence-based development of a diagnosis-dependent therapy planning system and its implementation in modern diagnostic software	Full paper not available
Kim et al., 2009	Predictive Modeling of Dental Pain Factors Using Neural Network Model	Article in foreign language without publisher provided translation
Khanna, 2010	Artificial intelligence: contemporary applications and future compass	Full paper not available
Lucey et al., 2010	Automatically detecting pain in video through facial action units	No implementations to orofacial healthcare
Mansor et al., 2014	Nonlinear Fuzzy Robust PCA Algorithm for Pain Decision Support System	No implementations to orofacial healthcare
Werner et al., 2014	Comparative learning applied to intensity rating of facial expressions of pain	No implementations to orofacial healthcare
Hussain et al., 2016	Development of an ANN optimized mucoadhesive buccal tablet containing flurbiprofen and lidocaine for dental pain	Non-human samples were used for data collection
Kim et al., 2016	The evaluation of implementing smart patient controlled analgesic pump with a different infusion rate for different time duration on postoperative pain management	Did not concern computer guided decision-making or pain management.
Seo, 2016	Smart syringe pumps for drug infusion during dental intravenous sedation	Did not concern computer guided decision-making
Rojas et al., 2017	Toward a functional near-infrared spectroscopy-based monitoring of pain assessment for nonverbal patients	No implementations to orofacial healthcare
Sornam, 2018	Logit-Based Artificial Bee Colony Optimization (LB-ABC) Approach for Dental Caries Classification Using a Back Propagation Neural Network	Book chapter describing a technique to improve accuracy of BPNN. The optimization technique was the primary focus without much description of the training design
Rojas et al., 2019	A Machine Learning Approach for the Identification of a Biomarker of Human Pain using fNIRS	No implementations to orofacial healthcare
Lin et al., 2019	Evaluation of computer-aided diagnosis system for detecting dental approximal caries lesions on periapical radiographs	Article in foreign language without publisher provided translation
Alshatrat et al, 2018	The use of immersive virtual reality for pain control during periodontal scaling and root planing procedures in dental hygiene clinic	Did not concern computer guided decision-making

Furman et al, 2009	Virtual reality distraction for pain control during periodontal scaling and root planing procedures	Did not concern computer guided decision-making
Kim et al, 2009	Predictive Modeling of Dental Pain Using Neural Network	Insufficient information on the machine learning design
Machado et al, 2018	Use of a Clinical Decision Support Tool for the Management of Traumatic Dental Injuries in the Primary Dentition by Novice and Expert Clinicians	Did not implement any form of machine learning. Questionnaire based possible outputs were provided.
Robertson, 2001	Knowledge-based system for structured examination, diagnosis and therapy in treatment of traumatised teeth	Did not implement the system within any orofacial healthcare scenarios
Won, 2017	Application of augmented reality for inferior alveolar nerve block anesthesia: A technical note	Did not concern computer guided decision-making

Section 2: MI-CLAIM Checklist

Article Title: Detecting caries lesions of different radiographic extension on bitewings using deep learning

Author (Year): Cantu et al (2020)

**MI-CLAIM Checklist**	
**Study design (Part 1)**	**Completed:**
The clinical problem in which the model will be employed is clearly detailed in the paper.	☑
The research question is clearly stated.	☑
The characteristics of the cohorts (training and test sets) are detailed in the text.	☑
The cohorts (training and test sets) are shown to be representative of real-world clinical settings.	☑
The state-of-the-art solution used as a baseline for comparison has been identified and detailed.	☑
**Data and optimization (Parts 2, 3)**	**Completed**
The origin of the data is described and the original format is detailed in the paper.	☑
Transformations of the data before it is applied to the proposed model are described.	☑
The independence between training and test sets has been proven in the paper.	☑
Details on the models that were evaluated and the code developed to select the best model are provided.	☑
Is the input data type structured or unstructured?<br>☐ Structured ☑ Unstructured	
**Model performance (Part 4)**	**Completed:**
The primary metric selected to evaluate algorithm performance (eg: AUC, F-score, etc) including the justification for selection, has been clearly stated.	☑
The primary metric selected to evaluate the clinical utility of the model (eg PPV, NNT, etc) including the justification for selection, has been clearly stated.	☑
The performance comparison between baseline and proposed model is presented with the appropriate statistical significance.	☑
**Model Examination (Parts 5)**	**Completed**
Examination Technique 1[a]	☐

Examination Technique 2[a]	☐
A discussion of the relevance of the examination results with respect to model/algorithm performance is presented.	☑
A discussion of the feasibility and significance of model interpretability at the case level if examination methods are uninterpretable is presented.	☑
A discussion of the reliability and robustness of the model as the underlying data distribution shifts is included.	☑
Common examination approaches based on study type:<br> For studies involving exclusively structured data coefficients and sensitivity analysis are often appropriate<br>* For studies involving unstructured data in the domains of image analysis or NLP: saliency maps (or equivalents) and sensitivity analysis are often appropriate	
**Reproducibility (Part 6): choose appropriate tier of transparency**	
Tier 1: complete sharing of the code	☐
Tier 2: allow a third party to evaluate the code for accuracy/fairness; share the results of this evaluation	☐
Tier 3: release of a virtual machine (binary) for running the code on new data without sharing its details	☐
Tier 4: no sharing	☐

PPV: Positive Predictive Value

NNT: Numbers Needed to Treat

[a] Common examination approaches based on study type: for studies involving exclusively structured data, coefficients and sensitivity analysis are often appropriate; for studies involving unstructured data in the domains of image analysis or natural language processing, saliency maps (or equivalents) and sensitivity analyses are often appropriate. Select 2 from this list or chose an appropriate technique, document each technique used on the appropriate line above.

Article Title: Development of a Deep Learning Algorithm for Periapical Disease Detection in Dental Radiographs

Author (Year): Endres et al (2020)

MI-CLAIM Checklist	
**Study design (Part 1)**	**Completed:**
The clinical problem in which the model will be employed is clearly detailed in the paper.	☑
The research question is clearly stated.	☐
The characteristics of the cohorts (training and test sets) are detailed in the text.	☑
The cohorts (training and test sets) are shown to be representative of real-world clinical settings.	☑
The state-of-the-art solution used as a baseline for comparison has been identified and detailed.	☑
**Data and optimization (Parts 2, 3)**	**Completed**
The origin of the data is described and the original format is detailed in the paper.	☑
Transformations of the data before it is applied to the proposed model are described.	☑
The independence between training and test sets has been proven in the paper.	☑
Details on the models that were evaluated and the code developed to select the best model are provided.	☐
Is the input data type structured or unstructured?<br>☐ Structured ☑ Unstructured	
**Model performance (Part 4)**	**Completed:**
The primary metric selected to evaluate algorithm performance (eg: AUC, F-score, etc) including the justification for selection, has been clearly stated.	☑
The primary metric selected to evaluate the clinical utility of the model (eg PPV, NNT, etc) including the justification for selection, has been clearly stated.	☑
The performance comparison between baseline and proposed model is presented with the appropriate statistical significance.	☑
**Model Examination (Parts 5)**	**Completed**
Examination Technique 1[a]	☐
Examination Technique 2[a]	☐

A discussion of the relevance of the examination results with respect to model/algorithm performance is presented.	☑
A discussion of the feasibility and significance of model interpretability at the case level if examination methods are uninterpretable is presented.	☑
A discussion of the reliability and robustness of the model as the underlying data distribution shifts is included.	☑
Common examination approaches based on study type:<br> For studies involving exclusively structured data coefficients and sensitivity analysis are often appropriate<br>* For studies involving unstructured data in the domains of image analysis or NLP: saliency maps (or equivalents) and sensitivity analysis are often appropriate	
**Reproducibility (Part 6): choose appropriate tier of transparency**	
Tier 1: complete sharing of the code	☐
Tier 2: allow a third party to evaluate the code for accuracy/fairness; share the results of this evaluation	☐
Tier 3: release of a virtual machine (binary) for running the code on new data without sharing its details	☐
Tier 4: no sharing	☐

PPV: Positive Predictive Value

NNT: Numbers Needed to Treat

[a] Common examination approaches based on study type: for studies involving exclusively structured data, coefficients and sensitivity analysis are often appropriate; for studies involving unstructured data in the domains of image analysis or natural language processing, saliency maps (or equivalents) and sensitivity analyses are often appropriate. Select 2 from this list or chose an appropriate technique, document each technique used on the appropriate line above.

Article Title: DeNTNet: Deep Neural Transfer Network for the detection of periodontal bone loss using panoramic dental radiographs

Author (Year): Kim et al (2019)

**MI-CLAIM Checklist**	
**Study design (Part 1)**	**Completed:** ✓
The clinical problem in which the model will be employed is clearly detailed in the paper.	✓ ☐
The research question is clearly stated.	☐
The characteristics of the cohorts (training and test sets) are detailed in the text.	☐ ✓
The cohorts (training and test sets) are shown to be representative of real-world clinical settings.	☐ ✓
The state-of-the-art solution used as a baseline for comparison has been identified and detailed.	☐ ✓
**Data and optimization (Parts 2, 3)**	**Completed**
The origin of the data is described and the original format is detailed in the paper.	☐ ✓
Transformations of the data before it is applied to the proposed model are described.	☐ ✓
The independence between training and test sets has been proven in the paper.	☐ ✓
Details on the models that were evaluated and the code developed to select the best model are provided.	☐ ✓
Is the input data type structured or unstructured?<br>☐ Structured ☐ Unstructured	
**Model performance (Part 4)**	**Completed:**
The primary metric selected to evaluate algorithm performance (eg: AUC, F-score, etc) including the justification for selection, has been clearly stated.	☐<br>(Not Justified)
The primary metric selected to evaluate the clinical utility of the model (eg PPV, NNT, etc) including the justification for selection, has been clearly stated.	☐<br>(Not Justified)
The performance comparison between baseline and proposed model is presented with the appropriate statistical significance.	☐<br>(No statistical significance test)
**Model Examination (Parts 5)**	**Completed**

Examination Technique 1[a]	☐
Examination Technique 2[a]	☐
A discussion of the relevance of the examination results with respect to model/algorithm performance is presented.	☐ ✓
A discussion of the feasibility and significance of model interpretability at the case level if examination methods are uninterpretable is presented.	☐ ✓
A discussion of the reliability and robustness of the model as the underlying data distribution shifts is included.	☐ ✓
Common examination approaches based on study type:<br> For studies involving exclusively structured data coefficients and sensitivity analysis are often appropriate<br>* For studies involving unstructured data in the domains of image analysis or NLP: saliency maps (or equivalents) and sensitivity analysis are often appropriate	
**Reproducibility (Part 6): choose appropriate tier of transparency**	
Tier 1: complete sharing of the code	☐
Tier 2: allow a third party to evaluate the code for accuracy/fairness; share the results of this evaluation	☐
Tier 3: release of a virtual machine (binary) for running the code on new data without sharing its details	☐
Tier 4: no sharing	☐

PPV: Positive Predictive Value

NNT: Numbers Needed to Treat

[a] Common examination approaches based on study type: for studies involving exclusively structured data, coefficients and sensitivity analysis are often appropriate; for studies involving unstructured data in the domains of image analysis or natural language processing, saliency maps (or equivalents) and sensitivity analyses are often appropriate. Select 2 from this list or chose an appropriate technique, document each technique used on the appropriate line above.

Article Title: Preliminary study on the application of deep learning system to diagnosis of Sjögren's syndrome on CT images

Author (Year): Kise et al (2019)

**MI-CLAIM Checklist**	
**Study design (Part 1)**	**Completed:**
The clinical problem in which the model will be employed is clearly detailed in the paper.	☑
The research question is clearly stated.	☑
The characteristics of the cohorts (training and test sets) are detailed in the text.	☑
The cohorts (training and test sets) are shown to be representative of real-world clinical settings.	☑
The state-of-the-art solution used as a baseline for comparison has been identified and detailed.	☑
**Data and optimization (Parts 2, 3)**	**Completed**
The origin of the data is described and the original format is detailed in the paper.	☑
Transformations of the data before it is applied to the proposed model are described.	☑
The independence between training and test sets has been proven in the paper.	☑
Details on the models that were evaluated and the code developed to select the best model are provided.	☑
Is the input data type structured or unstructured? ☐ Structured ☑ Unstructured	
**Model performance (Part 4)**	**Completed:**
The primary metric selected to evaluate algorithm performance (eg: AUC, F-score, etc) including the justification for selection, has been clearly stated.	☑
The primary metric selected to evaluate the clinical utility of the model (eg PPV, NNT, etc) including the justification for selection, has been clearly stated.	☑
The performance comparison between baseline and proposed model is presented with the appropriate statistical significance.	☑
**Model Examination (Parts 5)**	**Completed**
Examination Technique 1[a]	☐
Examination Technique 2[a]	☐

A discussion of the relevance of the examination results with respect to model/algorithm performance is presented.	☑
A discussion of the feasibility and significance of model interpretability at the case level if examination methods are uninterpretable is presented.	☑
A discussion of the reliability and robustness of the model as the underlying data distribution shifts is included.	☑
Common examination approaches based on study type:<br> For studies involving exclusively structured data coefficients and sensitivity analysis are often appropriate<br>* For studies involving unstructured data in the domains of image analysis or NLP: saliency maps (or equivalents) and sensitivity analysis are often appropriate	
**Reproducibility (Part 6): choose appropriate tier of transparency**	
Tier 1: complete sharing of the code	☐
Tier 2: allow a third party to evaluate the code for accuracy/fairness; share the results of this evaluation	☐
Tier 3: release of a virtual machine (binary) for running the code on new data without sharing its details	☐
Tier 4: no sharing	☐

PPV: Positive Predictive Value

NNT: Numbers Needed to Treat

[a] Common examination approaches based on study type: for studies involving exclusively structured data, coefficients and sensitivity analysis are often appropriate; for studies involving unstructured data in the domains of image analysis or natural language processing, saliency maps (or equivalents) and sensitivity analyses are often appropriate. Select 2 from this list or chose an appropriate technique, document each technique used on the appropriate line above.

Article Title: Usefulness of a deep learning system for diagnosing Sjögren's syndrome using ultrasonography images

Author (Year): Kise et al (2020)

**MI-CLAIM Checklist**	
**Study design (Part 1)**	**Completed:**
The clinical problem in which the model will be employed is clearly detailed in the paper.	☑
The research question is clearly stated.	☑
The characteristics of the cohorts (training and test sets) are detailed in the text.	☑
The cohorts (training and test sets) are shown to be representative of real-world clinical settings.	☑
The state-of-the-art solution used as a baseline for comparison has been identified and detailed.	☑
**Data and optimization (Parts 2, 3)**	**Completed**
The origin of the data is described and the original format is detailed in the paper.	☑
Transformations of the data before it is applied to the proposed model are described.	☑
The independence between training and test sets has been proven in the paper.	☑
Details on the models that were evaluated and the code developed to select the best model are provided.	☑
Is the input data type structured or unstructured?<br>☐ Structured ☑ Unstructured	
**Model performance (Part 4)**	**Completed:**
The primary metric selected to evaluate algorithm performance (eg: AUC, F-score, etc) including the justification for selection, has been clearly stated.	☑
The primary metric selected to evaluate the clinical utility of the model (eg PPV, NNT, etc) including the justification for selection, has been clearly stated.	☑
The performance comparison between baseline and proposed model is presented with the appropriate statistical significance.	☑
**Model Examination (Parts 5)**	**Completed**
Examination Technique 1[a]	☐
Examination Technique 2[a]	☐

A discussion of the relevance of the examination results with respect to model/algorithm performance is presented.	☑
A discussion of the feasibility and significance of model interpretability at the case level if examination methods are uninterpretable is presented.	☑
A discussion of the reliability and robustness of the model as the underlying data distribution shifts is included.	☑
Common examination approaches based on study type:<br> For studies involving exclusively structured data coefficients and sensitivity analysis are often appropriate<br>* For studies involving unstructured data in the domains of image analysis or NLP: saliency maps (or equivalents) and sensitivity analysis are often appropriate	
**Reproducibility (Part 6): choose appropriate tier of transparency**	
Tier 1: complete sharing of the code	☐
Tier 2: allow a third party to evaluate the code for accuracy/fairness; share the results of this evaluation	☐
Tier 3: release of a virtual machine (binary) for running the code on new data without sharing its details	☐
Tier 4: no sharing	☐

PPV: Positive Predictive Value

NNT: Numbers Needed to Treat

[a] Common examination approaches based on study type: for studies involving exclusively structured data, coefficients and sensitivity analysis are often appropriate; for studies involving unstructured data in the domains of image analysis or natural language processing, saliency maps (or equivalents) and sensitivity analyses are often appropriate. Select 2 from this list or chose an appropriate technique, document each technique used on the appropriate line above.

Article Title: Deep Learning for the Radiographic Detection of Periodontal Bone Loss

Author (Year): Krois et al (2019)

**MI-CLAIM Checklist**	
**Study design (Part 1)**	**Completed:**
The clinical problem in which the model will be employed is clearly detailed in the paper.	☐ ✓
The research question is clearly stated.	☐ ✓
The characteristics of the cohorts (training and test sets) are detailed in the text.	☐ ✓
The cohorts (training and test sets) are shown to be representative of real-world clinical settings.	☐ ✓
The state-of-the-art solution used as a baseline for comparison has been identified and detailed.	☐ ✓
**Data and optimization (Parts 2, 3)**	**Completed**
The origin of the data is described and the original format is detailed in the paper.	☐ ✓
Transformations of the data before it is applied to the proposed model are described.	☐ ✓
The independence between training and test sets has been proven in the paper.	☐ ✓
Details on the models that were evaluated and the code developed to select the best model are provided.	☐
Is the input data type structured or unstructured?<br>☐ Structured ☐ ✓ Unstructured	
**Model performance (Part 4)**	**Completed:**
The primary metric selected to evaluate algorithm performance (eg: AUC, F-score, etc) including the justification for selection, has been clearly stated.	☐ ✓
The primary metric selected to evaluate the clinical utility of the model (eg PPV, NNT, etc) including the justification for selection, has been clearly stated.	☐ ✓
The performance comparison between baseline and proposed model is presented with the appropriate statistical significance.	☐ ✓
**Model Examination (Parts 5)**	**Completed**
Examination Technique 1[a]	☐
Examination Technique 2[a]	☐
A discussion of the relevance of the examination results with respect to model/algorithm performance is presented.	☐ ✓

A discussion of the feasibility and significance of model interpretability at the case level if examination methods are uninterpretable is presented.	☐
A discussion of the reliability and robustness of the model as the underlying data distribution shifts is included.	☐ ✓
Common examination approaches based on study type:<br> For studies involving exclusively structured data coefficients and sensitivity analysis are often appropriate<br>* For studies involving unstructured data in the domains of image analysis or NLP: saliency maps (or equivalents) and sensitivity analysis are often appropriate	
**Reproducibility (Part 6): choose appropriate tier of transparency**	
Tier 1: complete sharing of the code	☐
Tier 2: allow a third party to evaluate the code for accuracy/fairness; share the results of this evaluation	☐
Tier 3: release of a virtual machine (binary) for running the code on new data without sharing its details	☐
Tier 4: no sharing	☐

PPV: Positive Predictive Value

NNT: Numbers Needed to Treat

[a] Common examination approaches based on study type: for studies involving exclusively structured data, coefficients and sensitivity analysis are often appropriate; for studies involving unstructured data in the domains of image analysis or natural language processing, saliency maps (or equivalents) and sensitivity analyses are often appropriate. Select 2 from this list or chose an appropriate technique, document each technique used on the appropriate line above.

Article Title: Deep-learning classification using convolutional neural network for evaluation of maxillary sinusitis on panoramic radiography

Author (Year): Murata et al (2018)

**MI-CLAIM Checklist**	
**Study design (Part 1)**	**Completed:**
The clinical problem in which the model will be employed is clearly detailed in the paper.	☑
The research question is clearly stated.	☑
The characteristics of the cohorts (training and test sets) are detailed in the text.	☑
The cohorts (training and test sets) are shown to be representative of real-world clinical settings.	☑
The state-of-the-art solution used as a baseline for comparison has been identified and detailed.	☑
**Data and optimization (Parts 2, 3)**	**Completed**
The origin of the data is described and the original format is detailed in the paper.	☑
Transformations of the data before it is applied to the proposed model are described.	☑
The independence between training and test sets has been proven in the paper.	☑
Details on the models that were evaluated and the code developed to select the best model are provided.	☑
Is the input data type structured or unstructured?<br>☐ Structured ☑ Unstructured	
**Model performance (Part 4)**	**Completed:**
The primary metric selected to evaluate algorithm performance (eg: AUC, F-score, etc) including the justification for selection, has been clearly stated.	☑
The primary metric selected to evaluate the clinical utility of the model (eg PPV, NNT, etc) including the justification for selection, has been clearly stated.	☑
The performance comparison between baseline and proposed model is presented with the appropriate statistical significance.	☑
**Model Examination (Parts 5)**	**Completed**
Examination Technique 1[a]	☐
Examination Technique 2[a]	☐

A discussion of the relevance of the examination results with respect to model/algorithm performance is presented.	☑
A discussion of the feasibility and significance of model interpretability at the case level if examination methods are uninterpretable is presented.	☑
A discussion of the reliability and robustness of the model as the underlying data distribution shifts is included.	☐
Common examination approaches based on study type:<br> For studies involving exclusively structured data coefficients and sensitivity analysis are often appropriate<br>* For studies involving unstructured data in the domains of image analysis or NLP: saliency maps (or equivalents) and sensitivity analysis are often appropriate	
**Reproducibility (Part 6): choose appropriate tier of transparency**	
Tier 1: complete sharing of the code	☐
Tier 2: allow a third party to evaluate the code for accuracy/fairness; share the results of this evaluation	☐
Tier 3: release of a virtual machine (binary) for running the code on new data without sharing its details	☐
Tier 4: no sharing	☐

PPV: Positive Predictive Value

NNT: Numbers Needed to Treat

[a] Common examination approaches based on study type: for studies involving exclusively structured data, coefficients and sensitivity analysis are often appropriate; for studies involving unstructured data in the domains of image analysis or natural language processing, saliency maps (or equivalents) and sensitivity analyses are often appropriate. Select 2 from this list or chose an appropriate technique, document each technique used on the appropriate line above.

Article Title: Deep Learning for Automated Detection of Cyst and Tumors of the Jaw in Panoramic Radiographs

Author (Year): Yang et al (2020)

MI-CLAIM Checklist	
**Study design (Part 1)**	**Completed:**
The clinical problem in which the model will be employed is clearly detailed in the paper.	☑ ✓
The research question is clearly stated.	☑ ✓
The characteristics of the cohorts (training and test sets) are detailed in the text.	☑ ✓
The cohorts (training and test sets) are shown to be representative of real-world clinical settings.	☑ ✓
The state-of-the-art solution used as a baseline for comparison has been identified and detailed.	☑ ✓
**Data and optimization (Parts 2, 3)**	**Completed**
The origin of the data is described and the original format is detailed in the paper.	☑ ✓
Transformations of the data before it is applied to the proposed model are described.	☑ ✓
The independence between training and test sets has been proven in the paper.	☑ ✓
Details on the models that were evaluated and the code developed to select the best model are provided.	☑ ✓
Is the input data type structured or unstructured?<br>☐ Structured ☐ Unstructured	
**Model performance (Part 4)**	**Completed:**
The primary metric selected to evaluate algorithm performance (eg: AUC, F-score, etc) including the justification for selection, has been clearly stated.	☑ ✓
The primary metric selected to evaluate the clinical utility of the model (eg PPV, NNT, etc) including the justification for selection, has been clearly stated.	☑ ✓
The performance comparison between baseline and proposed model is presented with the appropriate statistical significance.	☑ ✓
**Model Examination (Parts 5)**	**Completed**
Examination Technique 1[a]	☐
Examination Technique 2[a]	☐

A discussion of the relevance of the examination results with respect to model/algorithm performance is presented.	☑
A discussion of the feasibility and significance of model interpretability at the case level if examination methods are uninterpretable is presented.	☑
A discussion of the reliability and robustness of the model as the underlying data distribution shifts is included.	☑
Common examination approaches based on study type:<br> For studies involving exclusively structured data coefficients and sensitivity analysis are often appropriate<br>* For studies involving unstructured data in the domains of image analysis or NLP: saliency maps (or equivalents) and sensitivity analysis are often appropriate	
**Reproducibility (Part 6): choose appropriate tier of transparency**	
Tier 1: complete sharing of the code	☐
Tier 2: allow a third party to evaluate the code for accuracy/fairness; share the results of this evaluation	☐
Tier 3: release of a virtual machine (binary) for running the code on new data without sharing its details	☐
Tier 4: no sharing	☐

PPV: Positive Predictive Value

NNT: Numbers Needed to Treat

[a] Common examination approaches based on study type: for studies involving exclusively structured data, coefficients and sensitivity analysis are often appropriate; for studies involving unstructured data in the domains of image analysis or natural language processing, saliency maps (or equivalents) and sensitivity analyses are often appropriate. Select 2 from this list or chose an appropriate technique, document each technique used on the appropriate line above.

Section 3: Cochrane GRADE

Question: Should machine learning be used to diagnose pain related disorders in clinical dentistry?

Sensitivity	0.01 to 1.00
Specificity	0.01 to 1.00

Prevalences*	55.1%	74.5%	42%

Outcome	№ of studies (№ of patients)	Study design	Factors that may decrease certainty of evidence					Effect per 1,000 patients tested			Test accuracy CoE
			Risk of bias	Indirectness	Inconsistency	Imprecision	Publication bias	pre-test probability of 55.1%	pre-test probability of 74.5%	pre-test probability of 42%	
**True positives** (patients with pain related disorders)	6 studies 452 patients	cross-sectional (cohort type accuracy study)	not serious	not serious	serious [a,b]	not serious	all plausible residual confounding would reduce the demonstrated effect	6 to 551	7 to 745	4 to 420	⨁⨁⨁⨁ HIGH
**False negatives** (patients incorrectly classified as not having pain related disorders)								0 to 545	0 to 738	0 to 416	
**True negatives** (patients without pain related disorders)	6 studies 152 patients	cross-sectional (cohort type accuracy study)	serious [c,d]	not serious	not serious [e]	not serious	all plausible residual confounding would reduce the demonstrated effect	4 to 449	3 to 255	6 to 580	⨁⨁⨁⨁ HIGH
**False positives** (patients incorrectly classified as having pain related disorders)								0 to 445	0 to 252	0 to 574	

Explanations

* Prevalence analyses were made according to the following criteria:

1. 55.4% patients Typically had dental pain in the last 6 months
2. 74.5% patients attended the dental practice exclusively for pain
   (Kakoei S, Parirokh M, Nakhaee N, Jamshidshirazi F, Rad M, Kakooei S. Prevalence of toothache and associated factors: a population-based study in southeast iran. Iranian endodontic journal. 2013;8(3):123.)
3. 40-44% patients with dental pain were diagnosed with orofacial disorders in practice
   (Pau AK, Croucher R, Marcenes W. Prevalence estimates and associated factors for dental pain: a review. Oral Health Prev Dent. 2003 Jun 1;1(3):209)

a. Most data was obtained for machine learning purposes, therefore data in certain cases were intentionally screened to ensure larger or equal numbers of positive cases were present. While this may have been a necessity for the AI training purpose, it would add some degree of overall inconsistency when compared with the human reference standards

b. The cause of pain is directly affected by the prevalance of orofacial disease causing it. Some diseases have lower prevalance and therefore had to be marked up in order to train and test the machine learning process. Diagnostic outcomes may vary for such cases greatly when introduced into real world applications

c. A lower or equal number of negative cases were chosen in most studies for training and validation

d. A true randomized approach was not used when evaluating the performance

e. Periodontal diseases do not follow a dichotomous cut off and therefore multiple parameters exist for a single evaluation. As such, Kim et al and Krois et al's findings were not taken into account as several parameters (degree and level of bone loss) were evaluated for a single diagnostic analysis

Section 4: JBI-DTA Checklist

Title: Detecting caries lesions of different radiographic extension on bitewings using deep learning

JBI CRITICAL APPRAISAL CHECKLIST FOR DIAGNOSTIC TEST ACCURACY STUDIES

Reviewer________________________________ Date________________________

Author___Cantu et al___________________ Year__2020___ Record Number___1_____

	Yes	No	Unclear	Not applicable
1. Was a consecutive or random sample of patients enrolled?	☒	☐	☐	☐
2. Was a case control design avoided?	☒	☐	☐	☐
3. Did the study avoid inappropriate exclusions?	☒	☐	☐	☐
4. Were the index test results interpreted without knowledge of the results of the reference standard?	☐	☐	☐	☒
5. If a threshold was used, was it pre-specified?	☒	☐	☐	☐
6. Is the reference standard likely to correctly classify the target condition?	☒	☐	☐	☐
7. Were the reference standard results interpreted without knowledge of the results of the index test?	☐	☐	☐	☒
8. Was there an appropriate interval between index test and reference standard?	☐	☐	☐	☒
9. Did all patients receive the same reference standard?	☒	☐	☐	☐
10. Were all patients included in the analysis?	☒	☐	☐	☐

Overall appraisal: Include ☐ Exclude ☐ Seek further info ☐

Comments (Including reason for exclusion)

____

____

© JBI, 2020. All rights reserved. JBI grants use of these tools for research purposes only. All other enquiries should be sent to jbisynthesis@adelaide.edu.au.

Title: Development of a Deep Learning Algorithm for
Periapical Disease Detection in Dental Radiographs

JBI CRITICAL APPRAISAL CHECKLIST FOR DIAGNOSTIC TEST ACCURACY STUDIES

Reviewer ______________________ Date ______________________

Author Endres et al Year 2020 Record Number 2

	Yes	No	Unclear	Not applicable
1. Was a consecutive or random sample of patients enrolled?	☒	☐	☐	☐
2. Was a case control design avoided?	☐	☒	☐	☐
3. Did the study avoid inappropriate exclusions?	☒	☐	☐	☐
4. Were the index test results interpreted without knowledge of the results of the reference standard?	☐	☐	☐	☒
5. If a threshold was used, was it pre-specified?	☒	☐	☐	☐
6. Is the reference standard likely to correctly classify the target condition?	☒	☐	☐	☐
7. Were the reference standard results interpreted without knowledge of the results of the index test?	☐	☐	☐	☒
8. Was there an appropriate interval between index test and reference standard?	☐	☐	☐	☒
9. Did all patients receive the same reference standard?	☐	☐	☒	☐
10. Were all patients included in the analysis?	☒	☐	☐	☐

Overall appraisal: Include ☐ Exclude ☐ Seek further info ☐

Comments (Including reason for exclusion)

____

____

© JBI, 2020. All rights reserved. JBI grants use of these tools for research purposes only. All other enquiries should be sent to jbisynthesis@adelaide.edu.au.

Title: DeNTNet: Deep Neural Transfer Network for the detection of periodontal bone loss using panoramic dental radiographs

JBI CRITICAL APPRAISAL CHECKLIST FOR DIAGNOSTIC TEST ACCURACY STUDIES

Reviewer________________________ Date________________________

Author Kim et al Year 2019 Record Number 3

	Yes	No	Unclear	Not applicable
1. Was a consecutive or random sample of patients enrolled?	☑	☐	☐	☐
2. Was a case control design avoided?	☑	☐	☐	☐
3. Did the study avoid inappropriate exclusions?	☑	☐	☐	☐
4. Were the index test results interpreted without knowledge of the results of the reference standard?	☐	☐	☐	☑
5. If a threshold was used, was it pre-specified?	☑	☐	☐	☐
6. Is the reference standard likely to correctly classify the target condition?	☑	☐	☐	☐
7. Were the reference standard results interpreted without knowledge of the results of the index test?	☐	☐	☐	☑
8. Was there an appropriate interval between index test and reference standard?	☐	☐	☐	☑
9. Did all patients receive the same reference standard?	☑	☐	☐	☐
10. Were all patients included in the analysis?	☑	☐	☐	☐

Overall appraisal: Include ☐ Exclude ☐ Seek further info ☐

Comments (Including reason for exclusion)

____

____

© JBI, 2020. All rights reserved. JBI grants use of these tools for research purposes only. All other enquiries should be sent to jbisynthesis@adelaide.edu.au.

Title: Preliminary study on the application of deep learning system to diagnosis of Sjögren's syndrome on CT images

JBI CRITICAL APPRAISAL CHECKLIST FOR DIAGNOSTIC TEST ACCURACY STUDIES

Reviewer____ Date________________________________

Author____Kise et al___________________________ Year__2019_____ Record Number__4______

	Yes	No	Unclear	Not applicable
1. Was a consecutive or random sample of patients enrolled?	☒	☐	☐	☐
2. Was a case control design avoided?	☐	☒	☐	☐
3. Did the study avoid inappropriate exclusions?	☒	☐	☐	☐
4. Were the index test results interpreted without knowledge of the results of the reference standard?	☐	☐	☐	☒
5. If a threshold was used, was it pre-specified?	☒	☐	☐	☐
6. Is the reference standard likely to correctly classify the target condition?	☐	☐	☒	☐
7. Were the reference standard results interpreted without knowledge of the results of the index test?	☐	☐	☐	☒
8. Was there an appropriate interval between index test and reference standard?	☐	☐	☐	☒
9. Did all patients receive the same reference standard?	☒	☐	☐	☐
10. Were all patients included in the analysis?	☒	☐	☐	☐

Overall appraisal: Include ☐ Exclude ☐ Seek further info ☐

Comments (Including reason for exclusion)

____

____

© JBI, 2020. All rights reserved. JBI grants use of these tools for research purposes only. All other enquiries should be sent to jbisynthesis@adelaide.edu.au.

Title: Usefulness of a deep learning system for diagnosing Sjögren's syndrome using ultrasonography images

JBI CRITICAL APPRAISAL CHECKLIST FOR DIAGNOSTIC TEST ACCURACY STUDIES

Reviewer_______________________________________Date_______________________________

Author___Kise et al___________________________Year___2020___ Record Number___5___

	Yes	No	Unclear	Not applicable
1. Was a consecutive or random sample of patients enrolled?	☒	☐	☐	☐
2. Was a case control design avoided?	☐	☒	☐	☐
3. Did the study avoid inappropriate exclusions?	☒	☐	☐	☐
4. Were the index test results interpreted without knowledge of the results of the reference standard?	☐	☐	☐	☒
5. If a threshold was used, was it pre-specified?	☒	☐	☐	☐
6. Is the reference standard likely to correctly classify the target condition?	☐	☐	☒	☐
7. Were the reference standard results interpreted without knowledge of the results of the index test?	☐	☐	☐	☒
8. Was there an appropriate interval between index test and reference standard?	☐	☐	☐	☒
9. Did all patients receive the same reference standard?	☒	☐	☐	☐
10. Were all patients included in the analysis?	☒	☐	☐	☐

Overall appraisal: Include ☐ Exclude ☐ Seek further info ☐

Comments (Including reason for exclusion)

____

____

© JBI, 2020. All rights reserved. JBI grants use of these tools for research purposes only. All other enquiries should be sent to jbisynthesis@adelaide.edu.au.

Title: Deep Learning for the Radiographic Detection of Periodontal Bone Loss

JBI CRITICAL APPRAISAL CHECKLIST FOR DIAGNOSTIC TEST ACCURACY STUDIES

Reviewer________________ Date________________

Author Krois et al Year 2019 Record Number 6

	Yes	No	Unclear	Not applicable
1. Was a consecutive or random sample of patients enrolled?	☒	☐	☐	☐
2. Was a case control design avoided?	☒	☐	☐	☐
3. Did the study avoid inappropriate exclusions?	☒	☐	☐	☐
4. Were the index test results interpreted without knowledge of the results of the reference standard?	☐	☐	☐	☒
5. If a threshold was used, was it pre-specified?	☒	☐	☐	☐
6. Is the reference standard likely to correctly classify the target condition?	☐	☐	☒	☐
7. Were the reference standard results interpreted without knowledge of the results of the index test?	☐	☐	☐	☒
8. Was there an appropriate interval between index test and reference standard?	☐	☐	☐	☒
9. Did all patients receive the same reference standard?	☒	☐	☐	☐
10. Were all patients included in the analysis?	☒	☐	☐	☐

Overall appraisal: Include ☐ Exclude ☐ Seek further info ☐

Comments (Including reason for exclusion)

____

____

© JBI, 2020. All rights reserved. JBI grants use of these tools for research purposes only. All other enquiries should be sent to jbisynthesis@adelaide.edu.au.

Title: Deep-learning classification using convolutional neural network for evaluation of maxillary sinusitis on panoramic radiography

JBI CRITICAL APPRAISAL CHECKLIST FOR DIAGNOSTIC TEST ACCURACY STUDIES

Reviewer_______________ Date_______________

Author Murata et al Year 2018 Record Number 7

	Yes	No	Unclear	Not applicable
1. Was a consecutive or random sample of patients enrolled?	☒	☐	☐	☐
2. Was a case control design avoided?	☐	☒	☐	☐
3. Did the study avoid inappropriate exclusions?	☒	☐	☐	☐
4. Were the index test results interpreted without knowledge of the results of the reference standard?	☐	☐	☐	☒
5. If a threshold was used, was it pre-specified?	☒	☐	☐	☐
6. Is the reference standard likely to correctly classify the target condition?	☒	☐	☐	☐
7. Were the reference standard results interpreted without knowledge of the results of the index test?	☐	☐	☐	☒
8. Was there an appropriate interval between index test and reference standard?	☐	☐	☐	☒
9. Did all patients receive the same reference standard?	☒	☐	☐	☐
10. Were all patients included in the analysis?	☒	☐	☐	☐

Overall appraisal: Include ☐ Exclude ☐ Seek further info ☐

Comments (Including reason for exclusion)

____

____

© JBI, 2020. All rights reserved. JBI grants use of these tools for research purposes only. All other enquiries should be sent to jbisynthesis@adelaide.edu.au.

Title: Deep Learning for Automated Detection of Cyst and Tumors of the Jaw in Panoramic Radiographs

JBI CRITICAL APPRAISAL CHECKLIST FOR DIAGNOSTIC TEST ACCURACY STUDIES

Reviewer____________________ Date________________

Author Yang et al Year 2020 Record Number 8

	Yes	No	Unclear	Not applicable
1. Was a consecutive or random sample of patients enrolled?	☒	☐	☐	☐
2. Was a case control design avoided?	☐	☒	☐	☐
3. Did the study avoid inappropriate exclusions?	☒	☐	☐	☐
4. Were the index test results interpreted without knowledge of the results of the reference standard?	☐	☐	☐	☒
5. If a threshold was used, was it pre-specified?	☒	☐	☐	☐
6. Is the reference standard likely to correctly classify the target condition?	☒	☐	☐	☐
7. Were the reference standard results interpreted without knowledge of the results of the index test?	☐	☐	☐	☒
8. Was there an appropriate interval between index test and reference standard?	☐	☐	☐	☒
9. Did all patients receive the same reference standard?	☒	☐	☐	☐
10. Were all patients included in the analysis?	☒	☐	☐	☐

Overall appraisal: Include ☐ Exclude ☐ Seek further info ☐

Comments (Including reason for exclusion)

____

____

© JBI, 2020. All rights reserved. JBI grants use of these tools for research purposes only. All other enquiries should be sent to jbisynthesis@adelaide.edu.au.
