# Supplementary material for: Machine Learning and Intelligent Diagnostics in Dental and Orofacial Pain Management: A Systematic Review
Source: Pain Res Manag. 2021 Apr 26;2021:6659133. doi: 10.1155/2021/6659133 (PMC8093041; doi:10.1155/2021/6659133)
Supplement: Supplementary Materials — Supplementary Table S1: summary findings of literature for dental diseases. Supplementary Table S2: summary findings of literature for periodontal diseases. Supplementary Table S3: summary findings of literature for dental trauma and neuralgias. Supplementary Table S4: summary findings of the literature on cystic and neoplastic lesions. Supplementary Table S5: summary findings of the literature on glandular disorders. Supplementary Table S6: summary findings of the literature on bone and joint disorders. Supplementary Material S7. [file 6659133.f1.zip › 6659133.f1/Table 1. Dental and endodontics.docx]

**Supplementary Table S1:** Summary findings of literature for dental diseases

| **Author** | **Purpose of the study** | **Quantifications related to dental pain** | **Classification models used** | **Number of training models** | **Training model characteristics** | **Number of test models** | **Learning outcomes** | **Clinician’s role in the study design** | **Remarks** |
| --- | --- | --- | --- | --- | --- | --- | --- | --- | --- |
| Cantu et al, 2020 | Implementation of deep learning to detect caries progression from bitewing radiographs | extent and infiltration of proximal caries into dentinal tissue | U-net convoluted neural network with Intersection over Union (IoU) | 3293 proximal radiographs  (10 training cycles/epoch) | Radiographs of permanent dentitions with visually detectable varying proximal caries | 141 proximal radiographs | ***Machine outcomes***   - Accuracy = 0.80 - Sensitivity = 0.75 - Specificity = 0.83   ***Human outcomes***   - Accuracy = 0.71 - Sensitivity = 0.36 - Specificity = 0.91 | 7 experienced dentists were asked to identify extent and severity of caries for human comparison | - Deep learning provided more consistent diagnoses to proximal caries location and depth, and can be effective in identifying dentinal hypersensitivity - Dentists were less likely to detect incipient carious lesions |
| Hashem & Youssef, 2020 | Utilization of Big Data to predict dental infections | Quantified the exact infection present on the tooth | 1. Bacterial optimized recurrent neural network (BORNN) 2. Deep artificial neural network (DANN) 3. Genetic optimized neural network (GONN) 4. Adaptive neural network (ADNN) 5. big data analytic tool (Apache SAMOA) | 80 periapical radiographs | 1. Lab Archives BioMed Central Edition dataset radiographs of infected dental and oral tissue 2. Dental image repository dataset of 60,000 images used for tooth classification | 40 proximal radiographs | ***1) Multiscale segmented region***   - Accuracy = 0.95 - Sensitivity = 0.962 - Precision = 0.951 - Recall = 0.957   ***2) BORNN***   - Accuracy = 0.98 - Precision = 0.98 - Recall = 0.98 | - | - multiscale segemented region model for periapical radiographs encouraged accurate disease detection - BORNN had lowest error (0.189) and highest efficiency over the other models for dental infection detection |
| Hu et al, 2019 | Used machine learning to detect and localize hypersensitivity pain in real time from fNIR data of bilateral prefrontal and primary sensory cortices | Monitored cortical activities using optical neuro-imaging, augmented reality and neural network | 1. Convolutional neural networks (CNN) at 3,5,6 and 7 class layers 2. Artificial neural networks (ANN) on split data history blocks 3. Long short memory network (LSTM) | 1. ***Pain detection*** - 12 participants (180,580 data cubes) 2. ***Pain localization*** – 2 participants (30,820 data cubes) | fNIRS data of Human participants with dental hypersensitivity segmented into data cubes of pain and not pain | Same as training models. Evaluations were made on the ability to differentiate pain (13.24% of data) from no-pain (86.76%) | 1. ***Pain detection:***  - Accuracy = 0.80 (ANN) - Sensitivity = 0.41 (ANN with oversample) - Specificity = 0.896 (CNN-7)  1. ***Pain localization:***  - Accuracy = 0.74 (CNN-6) - Sensitivity = 0.54 (CNN-7) - Specificity = 0.86 (CNN-6) | - | - The authors mentioned that the highly sensitive data suggested that identifying ‘no-pain’ instead of pain would have been preferrable for machine learning as pain stimulus is lesser in quantity. - ANNs were more sensitive (0.41) but less specific (0.86) in reporting pain in real time. |
| Javed et al, 2019 | A neural network was set up to predict amount of bacteria remaining following caries removal | Streptococcus colony formation was observed pre- and post-caries removal. An ANN was built to predict post bacterial reinfections | Multi-layer perception artificial neural network with ‘ordinal-to-categorical’ encoding | ***Randomized data model:*** 45 streptococcus colony-forming-unit (CFU) lab results  (100 training cycles)  ***Cross validation***: 44 CFU results.  (25 training cycles) | Occlusal caries on vital, responsive deciduous molars | 1 sample of CFU (Leave-one-out cross validation) | - efficiency = 0.99 - Mean square error = 0.23 - Mean absolute % error = 4.96 - Train time = 67.30s | A dentist was asked to excavate occlusal caries using carbide bur, acrylic bur and spoon excavators. This served as the feeder data | A slower learning rate (0.0001) in the current study showed significantly better results compared to past studies with faster learning rates (0.005-0.5) |
| Liu et al, 2019 | Classified dental disease images to create a user-end interface to intelligently diagnose the source of pain and other symptoms | 1 MP CMOS sensor camera was used to record oral environments and isolate areas classified as dental disease | Mask Region-based convolutional neural network (RCNN) | 10,080 dental disease images (80%) acquired per frame from video | Dental images of occlusal caries, fluorosis, periodontitis, cracked tooth, calculus, plaque and missing space | 2520 disease images (20%) | ***Caries***   - Sensitivity = 0.98 - Specificity = 0.93 - Recognition rate = 0.90   ***Periodontitis***   - Sensitivity = 0.97 - Specificity = 0.95 - Recognition rate = 0.94   ***Cracked tooth***   - Sensitivity = 0.75 - Specificity = 0.99 - Recognition rate = 0.94 | 20 dentists were recruited to label the training library and screen for disease classification accuracy | Mask R-CNN was able to reliably classify dental diseases in the presence of environmental inferences such as saliva and dental spacing |
| Lee et al, 2018 | Designed a CNN to detect caries from radiographs | Localization of carious infection present on premolar and molar teeth | GoogLeNet v3 Inception v3 Convolutional neural network  (9 inception modules were used) | 2400 periapical radiographs  (50% carious, 50% non-carious)  32 batches per epoch (1000 epochs) | - Clear, denoised radiographs were collected, classified and labelled from electronic medical records (EMR) - Images were cropped to represent one permanent tooth per radiograph - Maxillary dentition images were flipped to resemble the mandibular arch | 600 periapical radiographs  (50% carious, 50% non-carious) | ***Premolar***   - Accuracy = 0.89 - Sensitivity = 0.84 - Specificity = 0.94   ***Molar***   - Accuracy = 0.88 - Sensitivity = 0.92 - Specificity = 0.84   ***Combined***   - Accuracy = 0.82 - Sensitivity = 0.81 - Specificity = 0.83 | Dentists labelled radiograph data that had clearly identifiable dental caries | - The authors suggest that the brightness, shadow and contrast on the radiographs greatly influence the prediction capabilities and therefore should not be solely relied on for caries detection - The training model characteristics vary across radiograph-based caries detection study and therefore yield varying degrees of machine accuracy |
| Berdouses et al, 2015 | Designed a classification system to label carious events based on ICDAS dental caries classification | Photographic, contrast enhanced images were segmented by clustering pre-cavitated and cavitated lesions | 1. J48 2. Random Tree 3. Random Forests (best result) 4. Support Vector Machines 5. Naïve Bayes | 103 digital images of occlusal surface (425 regions of interest) | 91 extracted teeth and 12 in-vivo non-hypoplastic teeth were photographed and pre-processed. 36 Features were extracted and evaluated for each pixel in the caries detected region | - | Random Forests on 3 classifications:  ***ICDAS 0***   - Sensitivity = 0.92 - Specificity = 1.00 - Precision = 0.99   ***ICDAS 1-2***   - Sensitivity = 0.77 - Specificity = 0.96 - Precision = 0.95   ***ICDAS 3-6***   - Sensitivity = 0.98 - Specificity = 0.84 - Precision = 0.74 | - Paediatric dentists labelled 35 images based on the ICDAS classification shown to them. - True positive and false negative outcomes were evaluated by the clinicians for the training dataset | - The intelligent system was built around clinicians’ diagnoses of photographs as opposed to radiographic or histological values - The system was able to precisely detect overlying changes to enamel (0-2) with lowered precision in detecting dentin involvement (3-6) |
| Rahman et al, 2014 | Designed a system to capture and classify light reflectance from cavitated tooth surface | A custom photodetector measured the amount of light reflectance and estimated the size of defect | Fuzzy logic and Single layer perceptron (SLP) neural network with hard limit transfer | 10 measures (63 epochs) for all training samples | 3 acrylic teeth and 2 natural canines were artificially cavitated. | 24 measures for acrylic and 13 measures for natural teeth | ***Classification accuracy***  Acrylic teeth = 1.0  Canine = 1.0 | - Drilled the defects onto the teeth simulating carious progression - Measured the extent of the defect using micro-meter scale | - SLP networks are useful for only two outputs that are linearly separable - Natural tooth decay produce overhangs which the current simulation did not take into consideration - The variations in tooth decay were also not taken into consideration due to the small training data size |
| Chattopadhyay et al, 2010 | Designed a learned model to process pain symptoms and predict the causative dental disease | 14 dental pain parameters were learned in combinations along with their possible disease outcome | 1. Naïve Bayesian Classifier (NBC) 2. Learned Bayes Classifier (LBC) | 1400 data (14*10*10) combination model | Dentists filled out questionnaires of pain characteristics for 40 clinical cases. The cases were categorized to fit the respective disease model | - | ***Dentinal Hypersensitivity***  Accuracy = 0.71  Sensitivity = 0.58  Specificity = 0.81  ***Acute Pulpitis***  Accuracy = 0.74  Sensitivity = 0.71  Specificity = 0.73  ***Apical Periodontitis***  Accuracy = 0.81  Sensitivity = 0.78  Specificity = 0.88  ***Chronic Pulpitis***  Accuracy = 0.78  Sensitivity = 0.48  Specificity = 0.93  ***Acute alveolar Abscess***  Accuracy = 0.64  Sensitivity = 0.64  Specificity = 0.96  ***Gingivitis***  Accuracy = 0.69  Sensitivity = 0.68  Specificity = 0.87 | 10 Dentists identified pain parameters and their corresponding dental diseases through questionnaires | Regression results from LBC classification analyses suggested only the following parameters to be significant for their respective 7 dental diseases:  clinical pain reproduction, quality of pain, tenderness of percussion, teeth drifting, facial swelling and gingival bleeding |
