# Supplementary material for: Machine Learning and Intelligent Diagnostics in Dental and Orofacial Pain Management: A Systematic Review
Source: Pain Res Manag. 2021 Apr 26;2021:6659133. doi: 10.1155/2021/6659133 (PMC8093041; doi:10.1155/2021/6659133)
Supplement: Supplementary Materials — Supplementary Table S1: summary findings of literature for dental diseases. Supplementary Table S2: summary findings of literature for periodontal diseases. Supplementary Table S3: summary findings of literature for dental trauma and neuralgias. Supplementary Table S4: summary findings of the literature on cystic and neoplastic lesions. Supplementary Table S5: summary findings of the literature on glandular disorders. Supplementary Table S6: summary findings of the literature on bone and joint disorders. Supplementary Material S7. [file 6659133.f1.zip › 6659133.f1/Table 2. Periodontology.docx]

**Supplementary Table S2:** Summary findings of literature for Periodontal diseases

| **Author** | **Purpose of the study** | **Quantification methods related to dental pain** | **Classification models used** | **Number of training models** | **Training model characteristics** | **Number of test models** | **learning outcomes** | **Clinician’s role in the study design** | **Remarks** |
| --- | --- | --- | --- | --- | --- | --- | --- | --- | --- |
| Chang et al, 2020 | Developed an intelligent system to detect the extent of periodontitis | CNN Detected Cementoenamel junction (CEJ) level and periodontal bone. The bone loss was then categorized using percentage rate analysis based on principal axes of teeth | Mask R-CNN based on Feature Pyramid Network (FPN) ResNet 101 | 518 panoramic images (90% from radiographs and 10% from data augmentation) | 340 contrast-normalized Panoramic radiographs of permanent dentition with defined periodontal bone level, CEJ level and teeth classification | 10 radiographs | ***CNN classification accuracy***   - Periodontal bone level = 0.92 - CEJ level = 0.87 - Teeth = 0.87   ***Mean absolute differences against clinicians***   - Maxillary periodontitis = 0.27 ±0.45 - Mandibular periodontitis = 0.23 ±0.43 | - Maxillofacial radiologists labelled areas of periodontal and alveolar bone loss in the training data - Radiologists provided diagnoses for comparison to the intelligent system | The system was developed based on the multidimensional grading and scaling of periodontitis. The proposed 2017 classification was centred around accurately identifying clinical attachment loss (CAL) or radiographic bone loss (RBL) |
| Endres et al, 2020 | Developed an intelligent system to detect periapical inflammation | The system detected and classified areas of radiolucencies based on clinically known characteristics of periapical diseases | U-Net convolutional neural network (CNN) | 2902 panoramic radiographs (872 were free of any diseases)  95 panoramic radiographs for learning validation | Good contrast, noise-free panoramic radiographs of following lesions: periapical inflammation, granuloma, cyst, tumour and osteomyelitis | 102 radiographs for versus comparison against clinicians | ***Surgeons’ diagnostic results***   - Precision = 0.69 - Sensitivity = 0.51   ***Machine learned model***   - Precision = 0.67 - Sensitivity = 0.51 | - 4 surgeons labelled the training data based on clinical experience - 24 maxillofacial surgeons were tested for their diagnostic accuracy on 102 radiographs | - On an average, 31% of the positive diagnoses made by the surgeons were inaccurate - The system was trained based visual clinical findings made by the surgeons. Therefore, man and machine had a high correlation (cor.=.72) in resulting outcomes |
| Setzer et al, 2020 | Developed an intelligent system to identify periapical lesions | A system detected and classified areas of radiolucencies from cone beamed computed tomography (CBCT) | U-Net convolutional neural network | 16 CBCT images (lesions were weighted 8X more based on clinical relevance) | 20 Normalized, limited field of view CBCTs with at least 1 periapical radiolucency. Endodontic status were disregarded during inclusion | 4 CBCT images | Sensitivity = 0.93  Specificity = 0.88 | 3 Radiologists and endodontists validated the accuracy of labelling on the training models | The sensitivity of the learned system was the same as the agreement outcomes (0.93) among the clinicians. This may indicate the clinicians’ experience to be a vital role in the deep learning outcomes. |
| Thanathornwong, 2020 | Developed a system to identify attachment levels of teeth to the surrounding periodontium | The system identified periodontal attachment losses >3mm with subsequent root exposures from panoramic radiographs | R-CNN based on ResNet architecture | 70 radiographs (70% of total data) | 100 panoramic radiographs with both healthy and periodontally compromised dentitions | 30 radiographs (10% for validation, 20% for accuracy) | Precision = 0.81  Sensitivity = 0.84  Specificity = 0.88 | 3 experienced Periodontologists localized teeth with attachment losses which they suspected to have periodontitis | Large intersection bounding boxes were used to isolate the entire area of suspected disease. This can make the outcomes less specific if used in crowded or deciduous dentitions. |
| Ekert et al, 2019 | Developed an intelligent system to identify periapical lesions | A system detected and classified areas of apical lesions from panoramic radiographs | 7-layer feed forward CNN based on Keras framework. | 2238 teeth segments from all 85 panoramic radiographs for 10 cycles. (base) | teeth segments shadowed by vertebrae were excluded from each radiograph. Quality of the radiographs were not considered. Upper segments were flippled 180 degrees prior to CNN learning | 341 teeth segments from all radiographs (base) | ***Identification of Apical lesions***  ***Incisors***   - Sensitivity = 0.55 - Specificity = 0.92   ***Canines***   - Sensitivity = 0.52 - Specificity = 0.96   ***Premolars***   - Sensitivity = 0.50 - Specificity = 0.90   ***Molars***   - Sensitivity = 0.80 - Specificity = 0.70 | 6 dentists labelled each of the radiographic segments based on apical lesion classification | There was only moderate agreement (k=0.48) between the dentists in labelling the data and the dentists did not revisit the images afterward. This indicates that there will be variations in machine learning outcomes if a similar study is performed by a different set of dentists.  This was seen in this study also, as when only images that scored k=1.00 were taken, machine learning accuracy (=0.95) increased significantly. |
| Kim et al, 2019 | Developed an intelligent system to localize periodontal bone loss (PBL) | A system detected and classified areas of apical lesions according to the offending tooth from panoramic radiographs | DeNTNet: Deep-CNN with region of interest (ROI) segmentation network, Auxiliary loss, transfer learning and ensembled network (of premolars and molars) | 11198 dental panoramic radiographs  Validation = 190 radiographs | 12179 radiographs obtained from each patient after initial diagnosis. No follow-up radiographs or repetitions were included | Test = 800 radiographs | ***DeNTNet***   - Sensitivity = 0.77 - Specificity = 0.95   ***Hygienists’ average***   - Sensitivity = 0.78 - Specificity = 0.92 | - 5 dental hygienists identified and labelled PBL lesions on the lesions - A maxillofacial surgeon further validated the labelling to improve reliability of learning model | Ablation study indicated that applying ROI can remove unnecessary classification radiographs and standardize the model. Additionally, ensembled network improved posterior tooth characterization associated to the bone loss. |
| Krois et al, 2019 | Developed an intelligent system to calculate periodontal bone loss (PBL) | Amount of bone loss was calculated in % from the distance between CEJ and alveolar crest by distance between CEJ and the apex | 7-layer feed-forward CNN based on Keras architecture | 1456 tooth segments from panoramic radiographs (10 epochs) | 85 Panoramic radiographs with tooth segments that did not overlap with the vertebrae | 353 segments from radiographs | ***CNN average***   - Accuracy = 0.81 - Sensitivity = 0.81 - Specificity = 0.81   ***Specialist average***   - Accuracy = 0.76 - Sensitivity = 0.92 - Specificity = 0.63 | - 3 examiners marked the PBL parameters on the radiograph - 6 endodontists and Periodontologists evaluated the test model for comparison with the learned system | - There was only moderate agreement (k=0.52) between the specialists in evaluating the test data - The learned system produced more accurate results in detecting PBL for both anterior and posterior teeth - The comparison evaluated 3 levels of boneloss; 20%, 25% and 30% with 20% considered as baseline reference - Both man and machine produced less accurate results for larger bone losses |
| Tiechmann et al, 2018 | An intelligent system to detect patient’s self-reported pain feedback during periodontal probing | Pain signals elicited during periodontal probing through ECG and PPG outputs were recorded using an in-house software and fed for machine learning | 1. Support Vector Machine (SVM 2. ANN 3. Random Forest Decision tree (best results) 4. K-Nearest neighbor (kNN) | 1370 pain signals from 40 individual patients | Healthy patients (BMI<30) with no neurological, cardiological, or endocrine disorders. The patient must have had periodontal cleaning 3 months prior and did not commit to substance abuse. | 195 pain samples from 7 patients | Accuracy = 0.70  Sensitivity = 0.71  Specificity = 0.70 | Clinicians probed periodontal pockets of patients to elicit recordable pain | - Patients’ response time to pain stimulus varies and this was taken into account in the current study by generating 5 pain intervals for each pain event - Pain was quantified using the following parameters:  heart-beat intervals (HBI), PPG amplitude, Surgical Pleth Index (SPI), Autonomic nervous system state index (ANSSi), Frequency spectral bins (FSB) and Levels of Discrete wavelength transforms (LDWT) |
| Meissner et al, 2005 | An intelligent system was developed to differentiate root surface from calculus based pathology during root scaling | An ultrasonic detection tool was designed to receive signals from piezo-driven hand scalers to classify anatomical and pathological structures | A fuzzy logic feature levels and learning system | 540 points on 70 teeth | Caries-free teeth that were extracted for periodontal therapy. The teeth were extracted, cleaned and preserved the teeth 3 weeks prior to the test | 200 points on 10 teeth | Accuracy = 0.82  Sensitivity = 0.87  Specificity = 0.76 | - | The system was able to correctly classify irrespective of scaler tip position (15-90 degree) and accidental lateral pressure |
