# Supplementary material for: Machine Learning and Intelligent Diagnostics in Dental and Orofacial Pain Management: A Systematic Review
Source: Pain Res Manag. 2021 Apr 26;2021:6659133. doi: 10.1155/2021/6659133 (PMC8093041; doi:10.1155/2021/6659133)
Supplement: Supplementary Materials — Supplementary Table S1: summary findings of literature for dental diseases. Supplementary Table S2: summary findings of literature for periodontal diseases. Supplementary Table S3: summary findings of literature for dental trauma and neuralgias. Supplementary Table S4: summary findings of the literature on cystic and neoplastic lesions. Supplementary Table S5: summary findings of the literature on glandular disorders. Supplementary Table S6: summary findings of the literature on bone and joint disorders. Supplementary Material S7. [file 6659133.f1.zip › 6659133.f1/Table 3. Trauma and Neuralgia.docx]

**Supplementary Table S3:** Summary findings of literature for dental trauma and neuralgias

| **Author** | **Purpose of the study** | **Quantification methods related to dental pain** | **Classification models used** | **Number of training models** | **Training model characteristics** | **Number of test models** | **learning outcomes** | **Clinician’s role in the study design** | **Remarks** |
| --- | --- | --- | --- | --- | --- | --- | --- | --- | --- |
| Fukuda et al, 2018 | Developed an intelligent system to detect vertical root fracture | A CNN was trained to localize vertical root fracture from panoramic radiographs | DetectNet CNN | 264 images of fractured teeth (1000 epochs) | 330 teeth from panoramic radiographs with clearly identifiable vertical root fracture | 66 images of fractured teeth | ***Average of maxillary and mandibular teeth***    ***Incisors***   - Sensitivity = 0.53 - Precision = 0.88   ***Premolar***   - Sensitivity = 0.70 - Precision = 0.95   ***Molar***   - Sensitivity = 0.70 - Precision = 0.96 | - 2 Maxillofacial radiologists and 1 endodontist selected the radiographs - 1 radiologist labelled the regions of fracture for the training dataset | - Anterior teeth scored poorer than posterior and was attributed to vertebrae overlapping within the radiograph - Maxillary teeth produced poorer classification results. This could be due to inadequate image pre-processing of the upper dentition segments. (Ekert et al., 2019) |
| Zhang et al, 2018 | Developed a system to predict facial swelling following 3^rd^ molar extractions | The system was fed data on patient’s; age, gender, physique, oral hygiene, 3^rd^ molar anatomy and relations to surrounding tissue. The swelling of each patient was then recorded as output data | Feed-forward, back-propagation CNN with 5-fold validation | 300 patients undergoing mandibular 3^rd^ molar extractions | The patients had no immunological or medication history. Post extraction swellings were labelled as 0 (<10mm), 0.5 (10-20mm) and 1.0 (>20mm) based on severity | 100 patients | ***Accuracy for levels of swelling***  Mild = 0.94  Moderate = 0.98  Severe = 0.94 | - 1 oral surgeon extracted all the teeth, provided post-extraction instructions, and followed up on the patients after 3 days - The surgeon physically measured facial swelling | - Increased training speed and reduced training cycles provided very high mean errors in predictive outcomes for facial swelling - Conjugate gradient technique improved convergence and learning accuracy - Reducing number of input parameters could yield better accuracy |
| McCartney et al, 2014 | Evaluated a questionnaire based intelligent diagnostic system to predict cause of facial pain | 22 binominal questions were asked for each participant to link to one of possible 9 causes | An in-house web based ANN | 607 patient questionnaires | All patients that presented with facial pain to a single centre and were diagnosed by experts.  Only trigeminal neuralgia (TN) type 1, TN Type 2 and Trigeminal neuropathic pain had large datasets (>50) | - | ***Trigeminal Neuralgia Type 1***   - Sensitivity = 0.92 - Specificity = 0.88   ***Trigeminal Neuralgia Type 2***   - Sensitivity = 0.63 - Specificity = 0.94   ***Trigeminal neuropathic pain***   - Sensitivity = 0.87 - Specificity = 0.95 | The clinical expert made the diagnoses for the patient data that was used to evaluate training outcome | The system was compared with the diagnoses made by a single expert. As seen in the previous studies, there are substantial amounts of disagreement between experts that can affect the comparison outcomes. Such effects were not taken into consideration here |
| Limonadi et al, 2006 | Developed a questionnaire based intelligent diagnostic system to predict cause of facial pain | 18 binominal questions were asked for each participant to link to one of possible 6 causes | An in-house web based ANN | 100 patient questionnaires (10,000 epochs) | All patients that presented with facial pain to a single centre and were diagnosed by experts.  Only trigeminal neuralgia (TN) type 1 and TN Type 2 had large datasets (>50) | 43 prospective patients | ***Trigeminal Neuralgia type 1***   - Sensitivity = 0.84 - Specificity = 0.83   ***Trigeminal Neuralgia type 2***   - Sensitivity = 0.50 - Specificity = 0.95 | The clinical expert made the diagnoses for the patient data that was used to evaluate training outcome | Atypical/idiopathic disorders (such as TN type 2) can produce a varying range of subjective clinical symptoms. Therefore, in the current study, they were less sensitive to questionnaire based machine learning methods. |
