# Supplementary material for: Machine Learning and Intelligent Diagnostics in Dental and Orofacial Pain Management: A Systematic Review
Source: Pain Res Manag. 2021 Apr 26;2021:6659133. doi: 10.1155/2021/6659133 (PMC8093041; doi:10.1155/2021/6659133)
Supplement: Supplementary Materials — Supplementary Table S1: summary findings of literature for dental diseases. Supplementary Table S2: summary findings of literature for periodontal diseases. Supplementary Table S3: summary findings of literature for dental trauma and neuralgias. Supplementary Table S4: summary findings of the literature on cystic and neoplastic lesions. Supplementary Table S5: summary findings of the literature on glandular disorders. Supplementary Table S6: summary findings of the literature on bone and joint disorders. Supplementary Material S7. [file 6659133.f1.zip › 6659133.f1/Table 4. Cystic and neoplastic lesions.docx]

**Supplementary Table S4:** Summary findings of literature on cystic and neoplastic lesions

| **Author** | **Purpose of the study** | **Quantification methods related to dental pain** | **Classification models used** | **Number of training models** | **Training model characteristics** | **Number of test models** | **learning outcomes** | **Clinician’s role in the study design** | **Remarks** |
| --- | --- | --- | --- | --- | --- | --- | --- | --- | --- |
| Kwon et al, 2020 | Developed a system to accurately diagnose various odontogenic cysts and tumours | Panoramic radiographs were labelled based on cortical margin and internal radiolucency | YOLO v3 CNN based on DarkNet-53 architecture | 946 panoramic radiographs with 12x data augmentation (8,000 epochs and learning rate of 0.001) | Panoramic radiographs of patients with ameloblastoma, dentigerous cysts, odontogenic keratocysts (OKC) and periapical cysts | 236 panoramic radiographs | ***Ameloblastoma***   - Accuracy = 0.94 - Sensitivity = 0.98 - Specificity = 1.00   ***Dentigerous cyst***   - Accuracy = 0.98 - Sensitivity = 0.99 - Specificity = 0.91   ***OKC***   - Accuracy = 0.94 - Sensitivity = 0.98 - Specificity = 0.92   ***Radicular cyst***   - Accuracy = 0.96 - Sensitivity = 0.99 - Specificity = 0.83 | 2 radiologists labelled histologically diagnosed cysts and tumors onto their corresponding radiographs | Cysts and tumours with cortical expansion in the upper jaw were said to increase the classification difficulty during deep learning primarily due to superimposition and presence of hollow structures such as nasal cavities and sinuses. |
| Watanabe et al, 2020 | Developed a system to differentiate radicular cysts from other lesions | Panoramic radiographs were used to classify radicular cyst, dentigerous cyst, odontogenic keratocyst and nasopalatine duct cyst | DetectNet CNN | 330 lesions (1000 epoch and 0.0001 learning rate) | Panoramic radiographs of 412 patients with histopathologically confirmed lesions and >10mm bone resorption | 71 lesions for validation and 35 lesions for testing | ***Anterior region cyst detection***   - Precision = 0.92 - Sensitivity = 1.00   ***Posterior region cyst detection***   - Precision = 0.88 - Sensitivity = 0.46   ***Radicular cyst classification***   - Precision = 0.87 - Sensitivity = 0.80 | 2 radiologists labelled and validated the radiographs using bounding boxes based on histopathology reports (ground truth) | - The lack of pretrained neural network and smaller training cycles in current study could have resulted in comparatively (Kwon et al, 2020) less favourable results and reduced learning rate. - The canine fossa and maxillary sinus affected the posterior region cyst detection sensitivity |
| Yang et al, 2020 | Developed a real-time detection and classification system for odontogenic tumors | Trained the system to detect Dentigerous cyst, odontogenic keratocyst and ameloblastoma from panoramic radiograph | CNN based on YOLO architecture | 1422 radiographs | 1603 panoramic radiographs of histopathologically confirmed lesions | 181 radiographs | ***Overall average detection***  ***Deep learning***   - Precision = 0.70 - Sensitivity = 0.68   ***Oral surgeons***   - Precision = 0.67 - Sensitivity = 0.67   ***General dentists***   - Precision = 0.65 - Sensitivity = 0.64 | 3 oral surgeons and 2 general dentists validated the intelligent system | - Specialists and general dentists demonstrated highest sensitivity for ameloblastoma and lowest sensitivity for odontogenic keratocysts - Deep learning was more precise and sensitive to odontogenic keratocysts |
