# Supplementary material for: Machine Learning and Intelligent Diagnostics in Dental and Orofacial Pain Management: A Systematic Review
Source: Pain Res Manag. 2021 Apr 26;2021:6659133. doi: 10.1155/2021/6659133 (PMC8093041; doi:10.1155/2021/6659133)
Supplement: Supplementary Materials — Supplementary Table S1: summary findings of literature for dental diseases. Supplementary Table S2: summary findings of literature for periodontal diseases. Supplementary Table S3: summary findings of literature for dental trauma and neuralgias. Supplementary Table S4: summary findings of the literature on cystic and neoplastic lesions. Supplementary Table S5: summary findings of the literature on glandular disorders. Supplementary Table S6: summary findings of the literature on bone and joint disorders. Supplementary Material S7. [file 6659133.f1.zip › 6659133.f1/Table 5. Glandular disorders.docx]

**Supplementary Table S5:** Summary findings of literature on glandular disorders

| **Author** | **Purpose of the study** | **Quantification methods related to dental pain** | **Classification models used** | **Number of training models** | **Training model characteristics** | **Number of test models** | **learning outcomes** | **Clinician’s role in the study design** | **Remarks** |
| --- | --- | --- | --- | --- | --- | --- | --- | --- | --- |
| Kise et al., 2020 | Developed a deep learning system to diagnose Sjogren Syndrome | Ultrasound Imaging of affected parotid (PG) and submandibular (SMG) gland morphology | VGG16 CNN with ImageNet dataset transfer learning | 160 patients (80 with Sjogren syndrome, 80 without) | 200 randomly grouped Ultrasound images of parotid and submandibular glands from patients with dry mouth. 50% were diagnosed with Sjogren syndrome while the remaining 50% were diagnosed free | 40 patients | ***PG Deep learning***   - Accuracy = 0.89 - Sensitivity = 0.90 - Specificity = 0.89   ***PG Radiologists***   - Accuracy = 0.77 - Sensitivity = 0.67 - Specificity = 0.86   ***SMG Deep learning***   - Accuracy = 0.84 - Sensitivity = 0.81 - Specificity = 0.87   ***SMG Radiologists***   - Accuracy = 0.72 - Sensitivity = 0.78 - Specificity = 0.66 | - Radiologists selected and processed the ultrasound images - 3 radiologists’ interpretation were compared against the learned system | Radiologists had better agreement when diagnosing the larger parotid glad (k= 0.65) than submandibular gland (k=0.51) |
| Kim et al, 2019 | Developed a system to detect maxillary sinusitis | The system was trained to identify sinusitis features from Paranasal Sinus (PNS) Xray using Water’s view radiograph. | Pretrained CNN models (VGG16, VGG19, ResNet-101) with a majority decision analysis (>90%) | 4860 PNS radiographs (50% sinusitis cases) with data augmentation | The images were compared against standard CT reference and defective images were excluded | 160 radiographs (Temporal test data) | ***Temporal dataset majority outcome***   - Accuracy = 0.94 - Sensitivity = 0.89 - Specificity = 0.99 | 2 radiologists labelled radiographs based on the presence or absence of sinusitis using bounding box | - The radiologists showed high agreement (k=0.85) when labelling sinusitis - The study utilized several deep learning models which only displayed results on which all models had high agreement |
| Kise et al, 2019 | Developed a system to identify salivary gland disorders | Computed tomography (CT) data to identify fatty degeneration within the salivary glands | AlexNet CNN with DIGITS library and Caffe framework | 400 segmented images from 40 CT scan data | CT scans were taken of patients with confirmed Sjogren Syndrome (50%). Another 50% healthy scans were used as control. Controls were ensured not to have any neoplasms or sialoliths | 100 segmented images from 10 CT scan data | ***Deep learning***   - Accuracy = 0.96 - Sensitivity = 1.00 - Specificity = 0.92   ***Experienced radiologists***   - Accuracy = 0.98 - Sensitivity = 0.99 - Specificity = 0.97   ***Inexperienced radiologists***   - Accuracy = 0.84 - Sensitivity = 0.78 - Specificity = 0.89 | 6 radiologists of varying experience evaluated the test data for comparison against the deep learned system | The findings suggested that a deep learned model had the diagnostic accuracy similar to a radiologist with 30 years of experience |
| Murata et al, 2018 | Developed a system to detect maxillary sinusitis | The system was trained to identify sinusitis features from panoramic radiographs | AlexNet CNN with DIGITS library and Caffe framework | 12000 patches (50% healthy and 50% sinusitis) obtained by data augmentation of 400 images (20 epochs) | Patients were screened for sinusitis hallmarks ex. Mucous thickening and pus discharge. The data was validated using CT scan as ground truth. The radiographs were cropped for regions of interest (patches) | 120 patches (50% healthy and 50% sinusitis) | ***Deep learning***   - Accuracy = 0.88 - Sensitivity = 0.86 - Specificity = 0.88   ***Experienced radiologists***   - Accuracy = 0.90 - Sensitivity = 0.90 - Specificity = 0.89   ***Inexperienced clinicians***   - Accuracy = 0.77 - Sensitivity = 0.78 - Specificity = 0.75 | 4 clinicians of varying experience evaluated the test data for comparison against the deep learned system | The findings suggested that a deep learned model had the diagnostic accuracy similar to a radiologist with 20 years of experience |
