# Supplementary material for: Machine Learning and Intelligent Diagnostics in Dental and Orofacial Pain Management: A Systematic Review
Source: Pain Res Manag. 2021 Apr 26;2021:6659133. doi: 10.1155/2021/6659133 (PMC8093041; doi:10.1155/2021/6659133)
Supplement: Supplementary Materials — Supplementary Table S1: summary findings of literature for dental diseases. Supplementary Table S2: summary findings of literature for periodontal diseases. Supplementary Table S3: summary findings of literature for dental trauma and neuralgias. Supplementary Table S4: summary findings of the literature on cystic and neoplastic lesions. Supplementary Table S5: summary findings of the literature on glandular disorders. Supplementary Table S6: summary findings of the literature on bone and joint disorders. Supplementary Material S7. [file 6659133.f1.zip › 6659133.f1/Table 6. Bone and joint disorders.docx]

**Supplementary Table S6:** Summary findings of literature on Bone and joint disorders

| **Author** | **Purpose of the study** | **Quantification methods related to dental pain** | **Classification models used** | **Number of training models** | **Training model characteristics** | **Number of test models** | **learning outcomes** | **Clinician’s role in the study design** | **Remarks** |
| --- | --- | --- | --- | --- | --- | --- | --- | --- | --- |
| Bianchi et al, 2020 | Developed an intelligent system for early detection of temporomandibular joint (TMJ) osteoarthritis | Used a combination of 52 normalized clinical, CBCT and biomolecular markers to predict early onset TMJ osteoarthritis | 1. Logistic regression 2. Extreme gradient boosting 3. Light gradient boosting 4. Random Forests | 96 patients (50% case, 50% control), each with 52 markers.  10 times 5 fold cross validation to create 1378 feature interactions | 96 Patients with no history of TMJ trauma or infection, pregnancy, congenital or cartilaginous disorders. Patients with >10 years of jaw pain and/or clinical symptoms of bone destruction were excluded | - | ***Deep learning for osteoarthritis***   - Accuracy = 0.82 - Sensitivity = 0.83 - Precision = 0.81   ***Deep learning for control***   - Sensitivity = 0.81 - Precision = 0.83 | Collected saliva and blood for biomarkers and took clinical history of the patients for data training | - The biomolecular markers used in the study were: 6ckine, Angiogenin, BDNF, CXCL16, ENA-78, MMP-3, MMP-7, OPG, PAI-1, TGFb1, TIMP-1, TRANCE, VE-Cadherin and VEGF - The clinical markers used were: Age and progression of TMJ pain, muscle soreness and mouth opening parameters - The radiomic markers used were: Energy, entropy, Emphases, bone volume, surface-volume ratio, trabecular thickness, separation, and number |
| Al Zubaidi, 2019 | Developed a system to detect osteoporosis from radiographs | Used several feature extraction methods to identify osteoporosis radiographic findings from panoramic radiographs | Self-Organizing Map (SOM) and Linear Vector Quantization (LVQ) ANN with   1. Gabor filter 2. Edge orientation histogram 3. Haar wavelet 4. Steerable filter | 575 panoramic radiographs (267 osteoporosis and 308 control) | 575 panoramic xrays. 267 were diagnosed osteoporosis based on Dual Energy Xray Absorption (DEXA) | - | ***Gabor-based model***   - Accuracy = 0.93 - Sensitivity = 0.97 - Specificity = 0.86 | - | The authors found intensity histogram, entropy, mean,  standard deviation, contrast, energy, and homogeneity feature extractors to produce poor accuracy in classifying osteoporosis from radiographs |
| Dumast et al, 2018 | Proposed a web-based system to aid clinicians in detecting TMJ osteoarthritis | Used CBCT clinical data, radiomics and biomolecular markers to predict early onset TMJ osteoarthritis | Shape-VariationAnalyzer (SVA) CNN based on Tensorflow library | 426 condyle mesh  Data augmented using Perlin noise and 50 epochs | 293 condyles were surface modelled from CBCT data  (154 with diagnosed osteoarthritis and 105 controls) | 34 condyle mesh from 17 patients with pain <5 years | classification of the degree of degeneration agreed (0.91) with the clinicians’ consensus | Clinicians diagnosed the cases prior to CBCT data collection  Nurses collected the serum samples  Clinicians classified the shapes in the CBCT data | - 17 protein biomarkers were collected: 6ckine, ANG, BDNF, CXCL16, ENA-78, GM-CSF, IFNγ, IL-1α, IL-6, MMP3, MMP-7, PAI-1, TGFβ1, TIMP-1, TNFα, VE-Cadherin and VEGF - Clinical evaluation found correlation (0.905) between current pain experienced by patients with osteoarthritis and the worst pain experienced in last 6 months |
| Nam et al, 2018 | Developed a system to identify TMJ disorder mimicking syndrome | Used informatics text mining to implement predictive clinical modelling to separate TMJ disorder mimicking syndromes from genuine TMJ disorders | Text mining and recursive partitioning | 29 diagnosed cases (case)  290 TMJ disorder patients (control) | - 29 diagnosed cases of TMJ mimicking disorder from 2001 to 2016 - 290 patients with TMJ disorder | - | - Accuracy = 0.96 - Specificity = 0.99 - Sensitivity = 0.69 | Dentists validated and differentiated between the two disease groups | - Inflammation and infections (abscess and pericoronitis) were found to be the most common cause (44%) ofTMJ disorder mimicking syndrome - Mimicking syndrome saw greater maximum mouth opening (26.5mm) than TMJ disorder |
| Bas et al, 2012 | Designed a system to predict TMJ disk displacement based on clinical symptoms | Clinical symptoms were characterized based on unilateral and bilateral anterior disk displacements with/without reduction | ANN with back-propagation using Visual Basic 6.0 | 161 patients | Patients with diagnosed with TMJ disk disorder. | 58 patients | ***Unilateral anterior disk displacement with reduction***   - Sensitivity = 0.80 - Specificity = 0.95   ***Unilateral anterior disk displacement without reduction***   - Sensitivity = 0.69 - Specificity = 0.91   ***Bilateral anterior disk displacement with reduction***   - Sensitivity = 1.00 - Specificity = 0.89   ***Bilateral anterior disk displacement without reduction***   - Sensitivity = 0.37 - Specificity = 1.00 | 1 oral surgeon categorized data based on the clinical symptoms exhibited for disk displacement | Diagnosis was made based on ‘Research Diagnostic Criteria of Temporomandibular  Disorder guidelines’ |
